# Supplementary material for: Comparative analysis reveals within-population genome size variation in a rotifer is driven by large genomic elements with highly abundant satellite DNA repeat elements
Source: BMC Biol. 2021 Sep 16;19:206. doi: 10.1186/s12915-021-01134-w (PMC8447722; doi:10.1186/s12915-021-01134-w)
Supplement: Supplementary file 1 — Additional file 1. Supplementary figures and tables. [file 12915_2021_1134_MOESM1_ESM.docx]

## Additional file 1 for:

**Genome structure of *Brachionus asplanchnoidis*, a Eukaryote with intrapopulation variation in genome size**

Stelzer, C.P., Blommaert J., Waldvogel A.M., Pichler M., Hecox-Lea, B. & Mark Welch D.B.

Correspondence to: [claus-peter.stelzer@uibk.ac.at](mailto:claus-peter.stelzer@uibk.ac.at)

## This file (Additional file 1) includes:

Supplementary results

Figures S1 to S19

Tables S1 to S11

**Other data provided as Additional files:**

Additional file 2 (VBCF report on genome assembly and contaminant filtering; pdf-file)

Additional file 3 (Summary of short-read preprocessing and fastqc-reports; xlsx-file)

Additional file 4 (Kmer-based analysis of cleaned Illumina reads, xlsx-file)

Additional file 5 (Ranges of all CNVs across the *B. asplanchnoidis* genome; csv-file)

Additional file 6 (Detailed information on top-36 contributing repeat elements; xlsx-file)

Additional file 7 (Repeat profile, Gene density, and CNVs of the 50 largest contigs; html-file)

Additional file 8 (Combined library of curated and uncurated repeat elements; fasta-file)

Additional file 9 (Input parameters of the thermoalign pipeline; txt-file)

**Data submitted to public databases:**

The raw reads, the genome assembly, and the gene annotation files have been submitted to

- Assembly
- Gene annotation files

**Supplementary results**

In total, we obtained 265.6 Gbp of Illumina short-read data from the 15 different *B. asplanchnoidis* clones. After all preprocessing steps had been completed, this amount was reduced to 194.3 Gbp. Total alignment rates to the reference genome improved from 90.9-98.4%, after quality-filtering only, to 94.1-98.4%, after contaminant removal (**Additional file 3**) Contamination rates were variable among the different libraries (**Fig. S1**). Contaminant DNA mostly derived from bacteria, of which *Pseudomonas toyotensis* was the only bacterial genome that could be identified with our metagenomic assembly-based approach. Among the libraries 3-30% of the unmapped reads could be assigned to other microbial contaminants. Contamination by the food algae *Tetraselmis* was low to absent depending on library (usually less than 1% of unmapped reads), and protozoan contamination was below the detection threshold in all libraries. After preprocessing, the genome coverages ranged between 9.4-79.0-fold, if the Gbp sequence of each library is expressed as multiples of the 230.1Mb *B. asplanchnoidis* reference genome, with the majority of libraries being above 20-fold (**Additional file 3**). Quality control using fastqc [1] indicated substantial improvements in most parameters in the course of the pre-processing pipeline, with the notable exception of GC-content (**Additional file 3**)

**
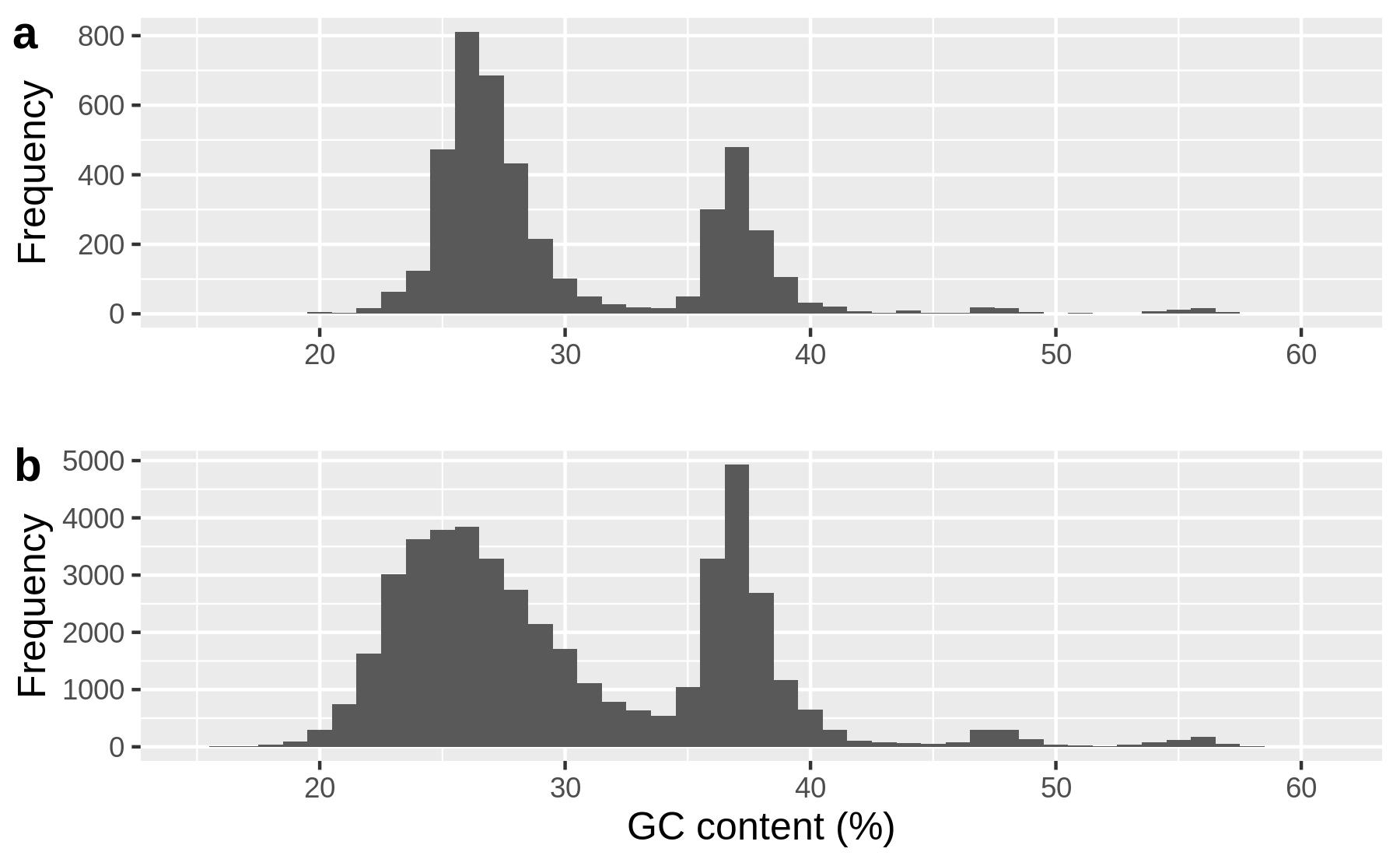
**

**Fig. S1:** GC-distribution in the reference assembly. **a** distribution based on 50kbp-windows, **b** distribution based on 5kbp-windows.


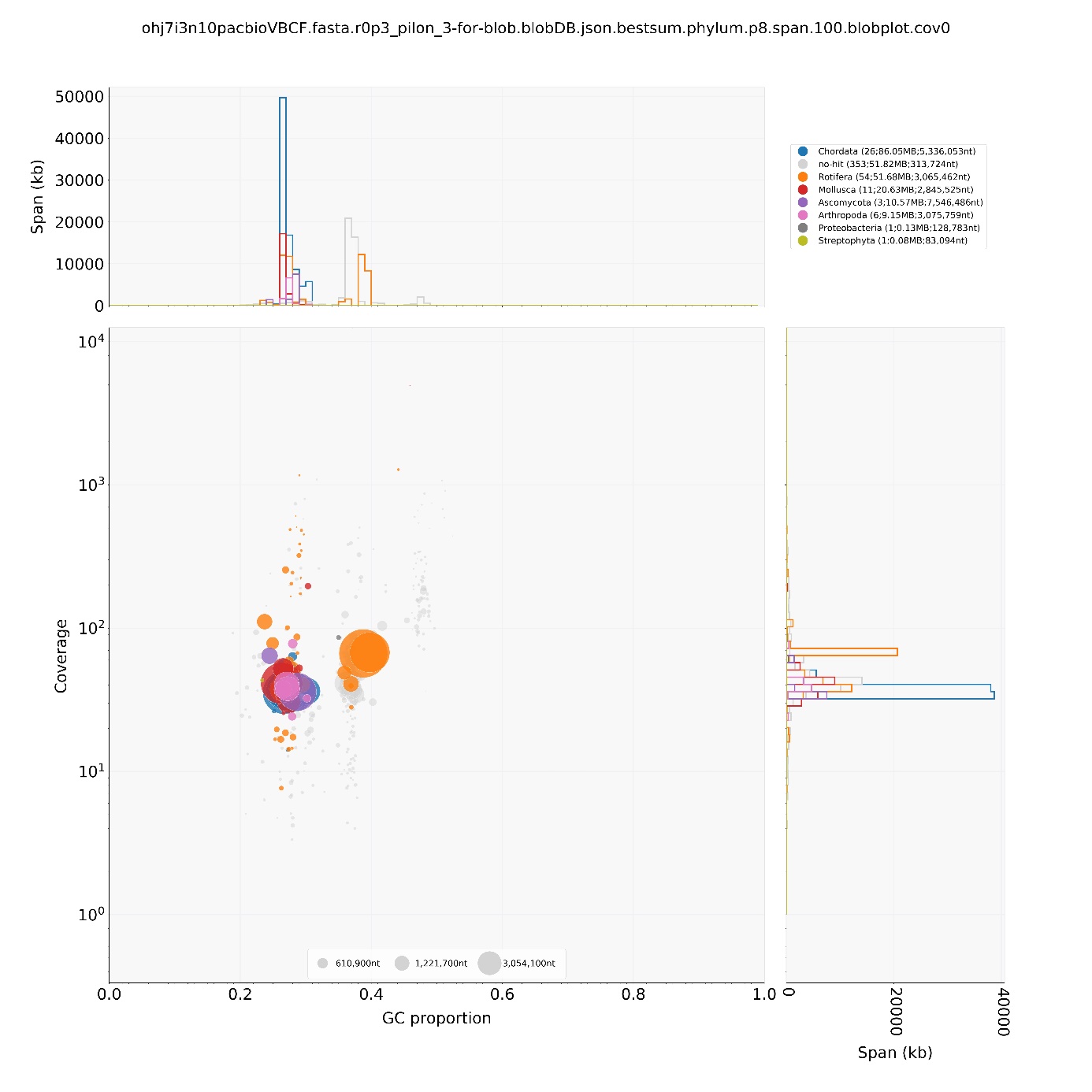


**Fig. S2: Blobplot of cleaned OHJ7i3n10 reads against reference genome.** Clone OHJ7i3n10 is the clone used in the reference genome assembly (see **Table 1**)


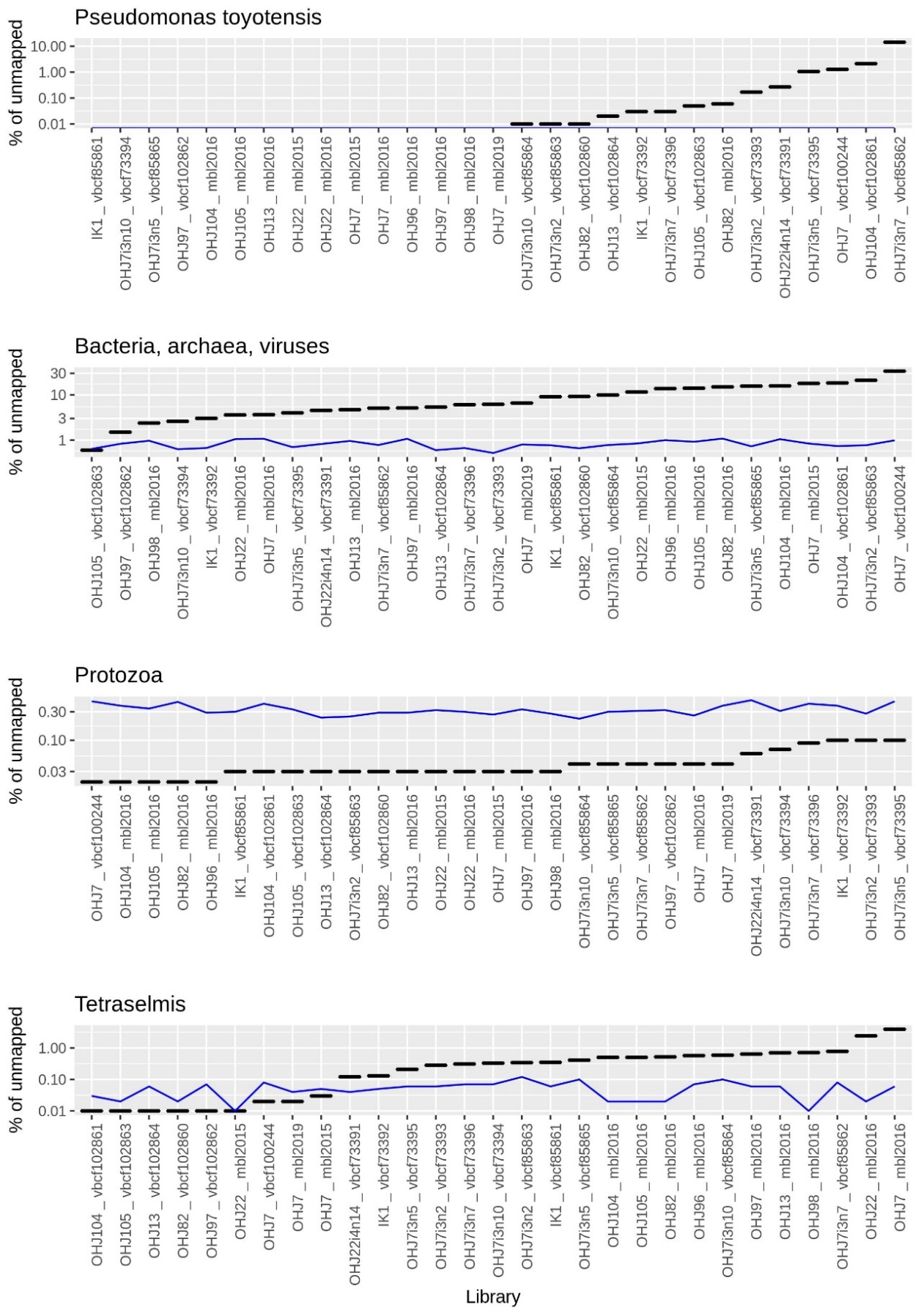


**Fig. S3: Contamination rates of short-read libraries (before contaminant removal).** Horizontal dashes indicate the percentage of reads in the unmapped-reads fraction (i.e., quality-trimmed reads that did *not* align to the reference genome in the first alignment) that could be assigned to four classes of contaminants: *Pseudomonas toyotensis*, (b) other bacteria, archaea, viruses, (c) protozoan contaminants, and (d) *Tetraselmis* (food algae). The blue line indicates the “false-discovery rate”, which was obtained by applying the same pipeline to the mapped-reads fraction of each library.


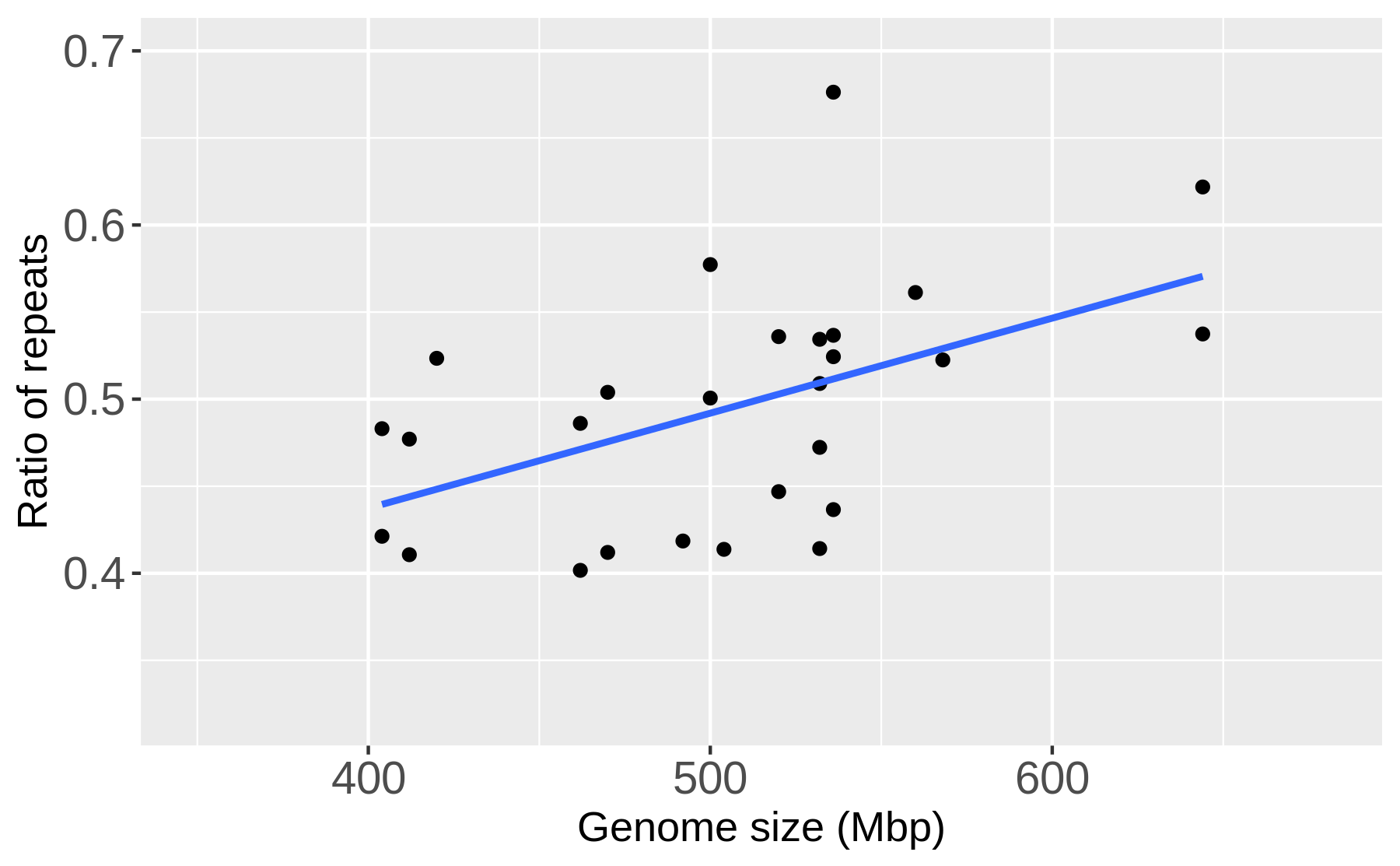


**Fig. S4: Genome size (2C, as measured by flow cytometry) versus ratio of repeats**, a fitted parameter of findGSE [2]. The two variables are significantly correlated with each other (Pearson correlation test, *r*=0.526, *p*=0.004). Genome size estimates were taken from [3].


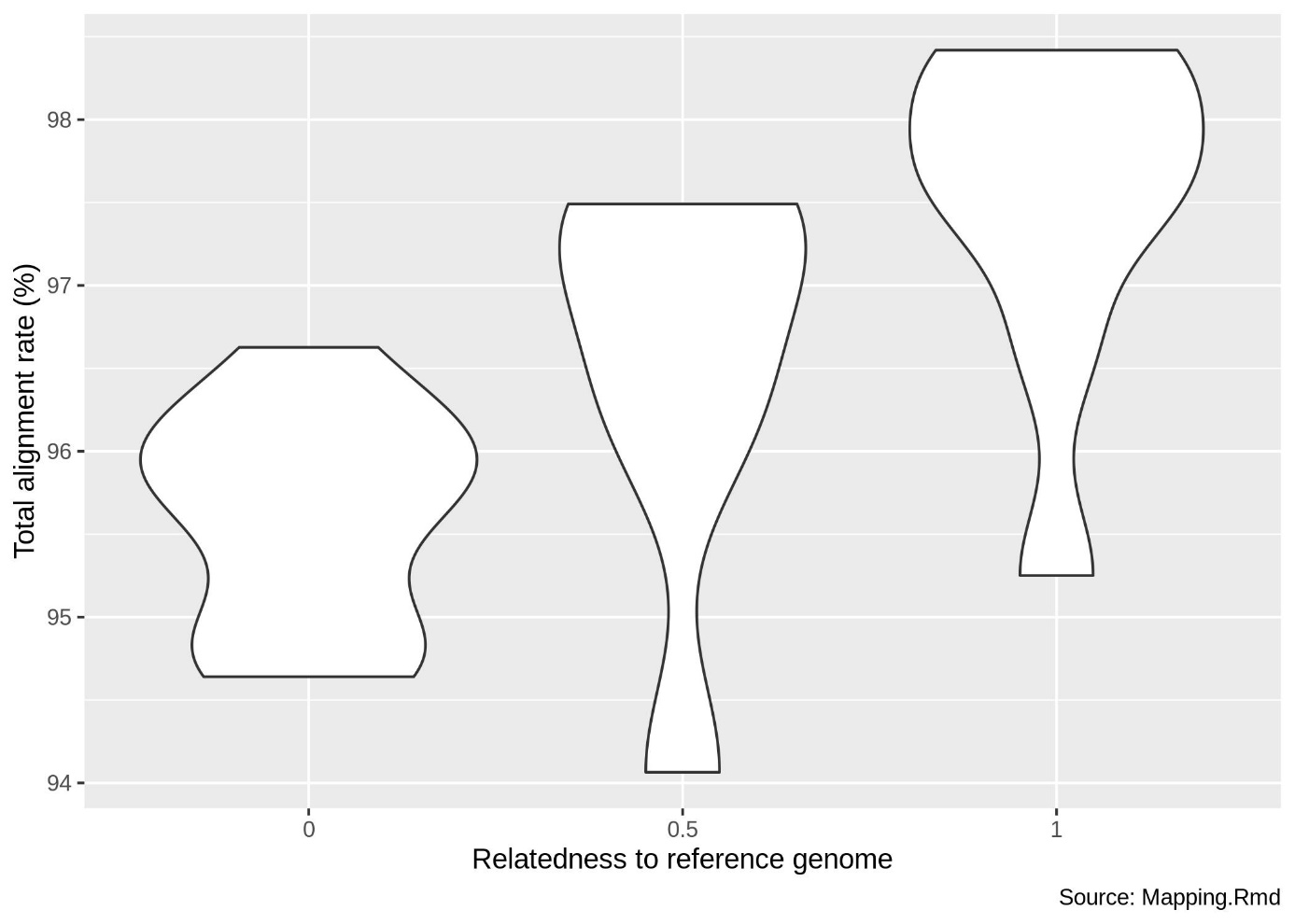


**Fig. S5: Total alignment rate *vs*. relatedness of DNA source to the reference genome.** Relatedness was assumed to be 1 for all clones within the OHJ7i3-selfed line, 0.5 for the inbred line cross (IK1) and the natural ancestor of the inbred line (OHJ7), and 0 for all other clones which hatched from the natural population.


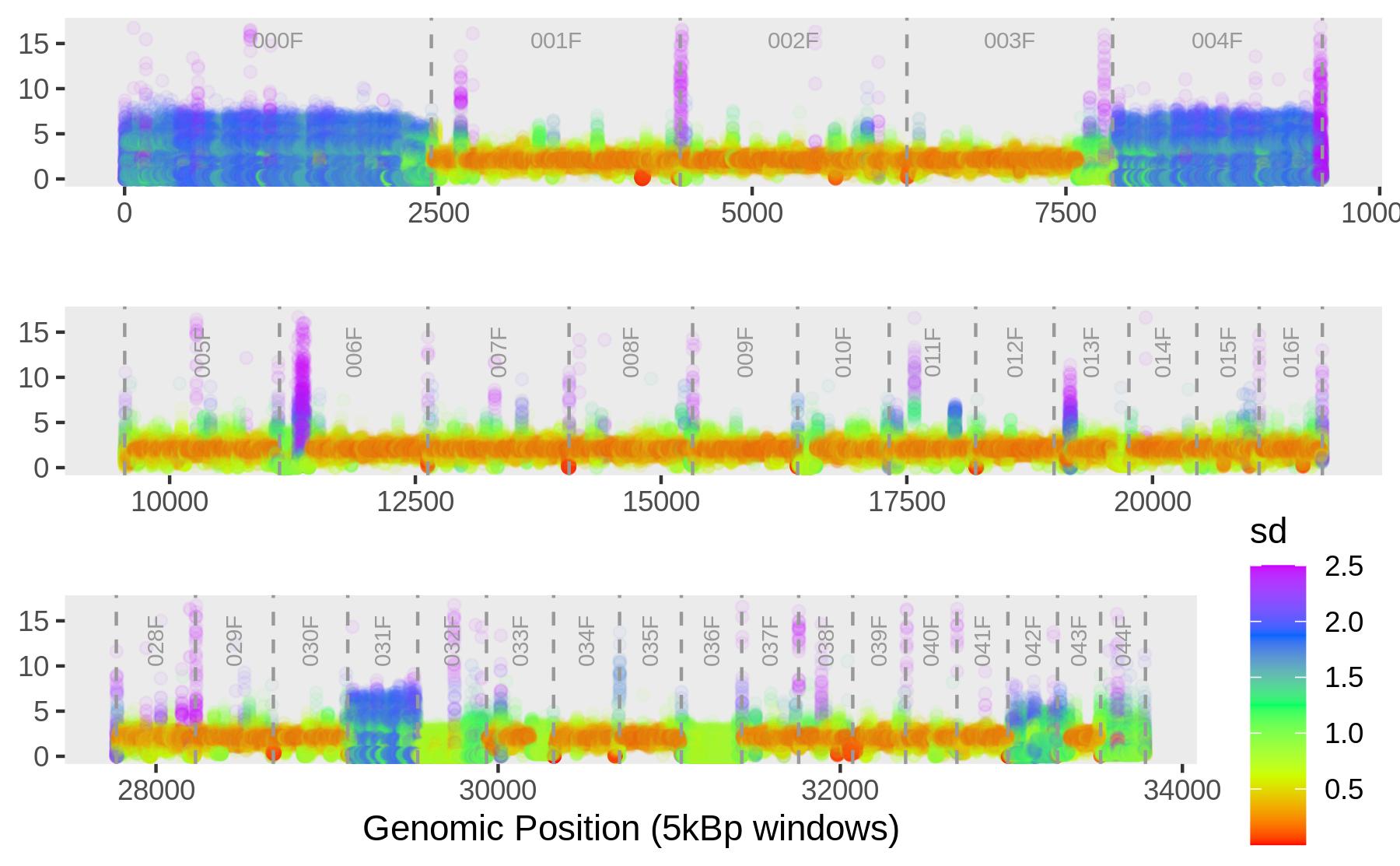


**Fig. S6:** **Coverage and coverage variation along representative contigs of the *B. asplanchnoidis* genome (5kBp resolution)**. Each circle represents a 5kBp window of one of the 31 sequencing libraries. Coverage (y-axis) was normalized by dividing the per-bp coverage of each window by 1/2 of the (mean) exon coverage of the respective library. Contig borders are indicated by vertical dashed lines, and contig IDs are listed on the top. All contigs displayed here span ~140 Mbp in total, i.e., about 60% of the assembly. Variation across clones/libraries is indicated by color (based on standard deviations). Standard deviations larger than 2.5 (about 4% of the data) had to be capped to a value 2.5, to allow colored visualization.


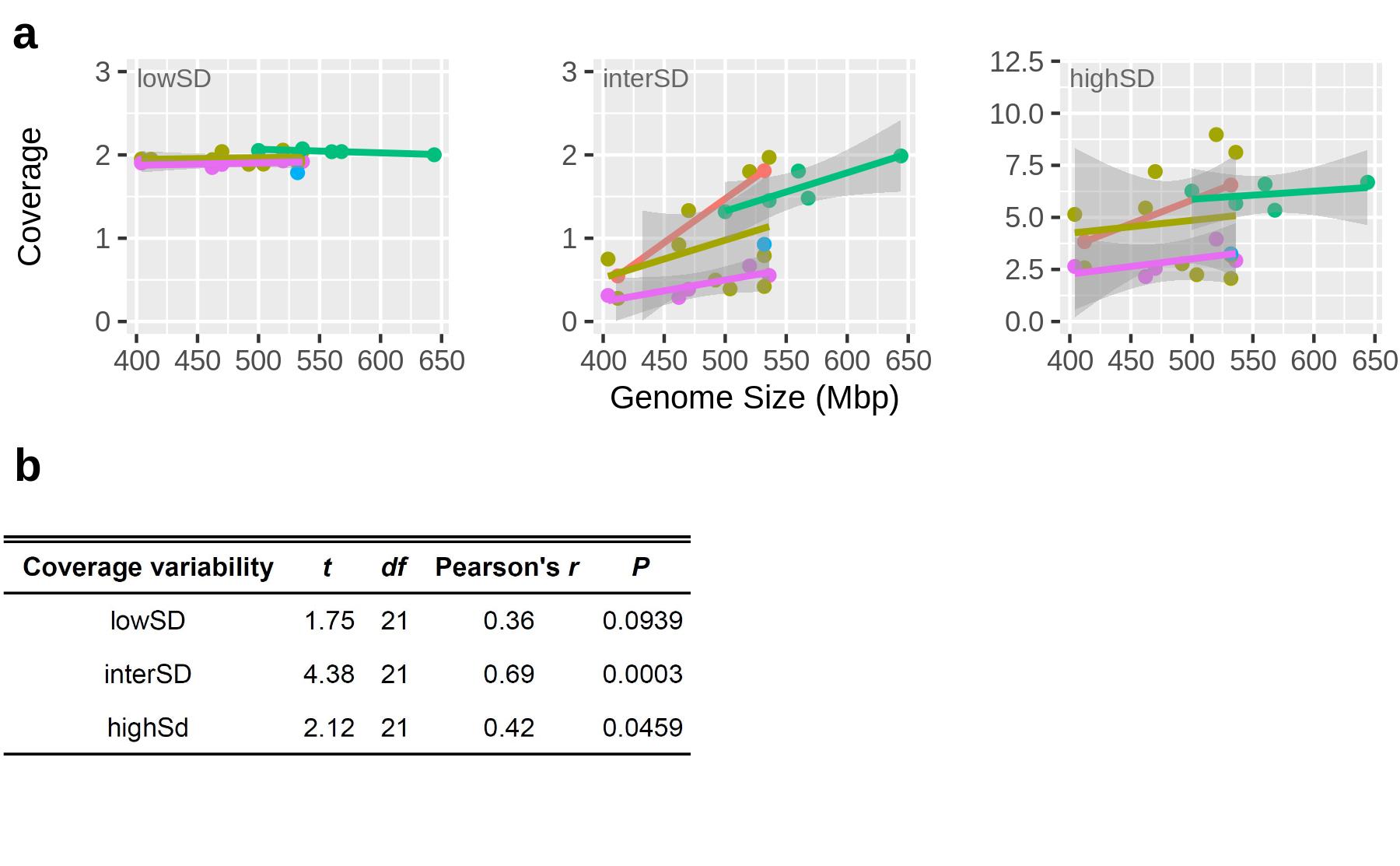


**Fig. S7: Genomic regions of elevated coverage variability are responsible for genome size variation.** This figure shows the same analysis as in Fig. 4 but it excludes the library preps “C” (c.f., Tab. S4), which showed considerably higher coverage and higher GC content in interSD and highSD regions than the other libraries. **a** Mean coverage versus genome size [flow cytometry data on genome size was taken from 3]. Dots represent the mean coverage per library. Colors indicate co-prepared libraries (as in **Tab. S4**): Orange = A, Pink = B, Green = D, Blue= E, Gold = F. Shaded regions are the 95% confidence intervals. **b** partial correlations between the variables ‘mean coverage’ and ‘genome size’.


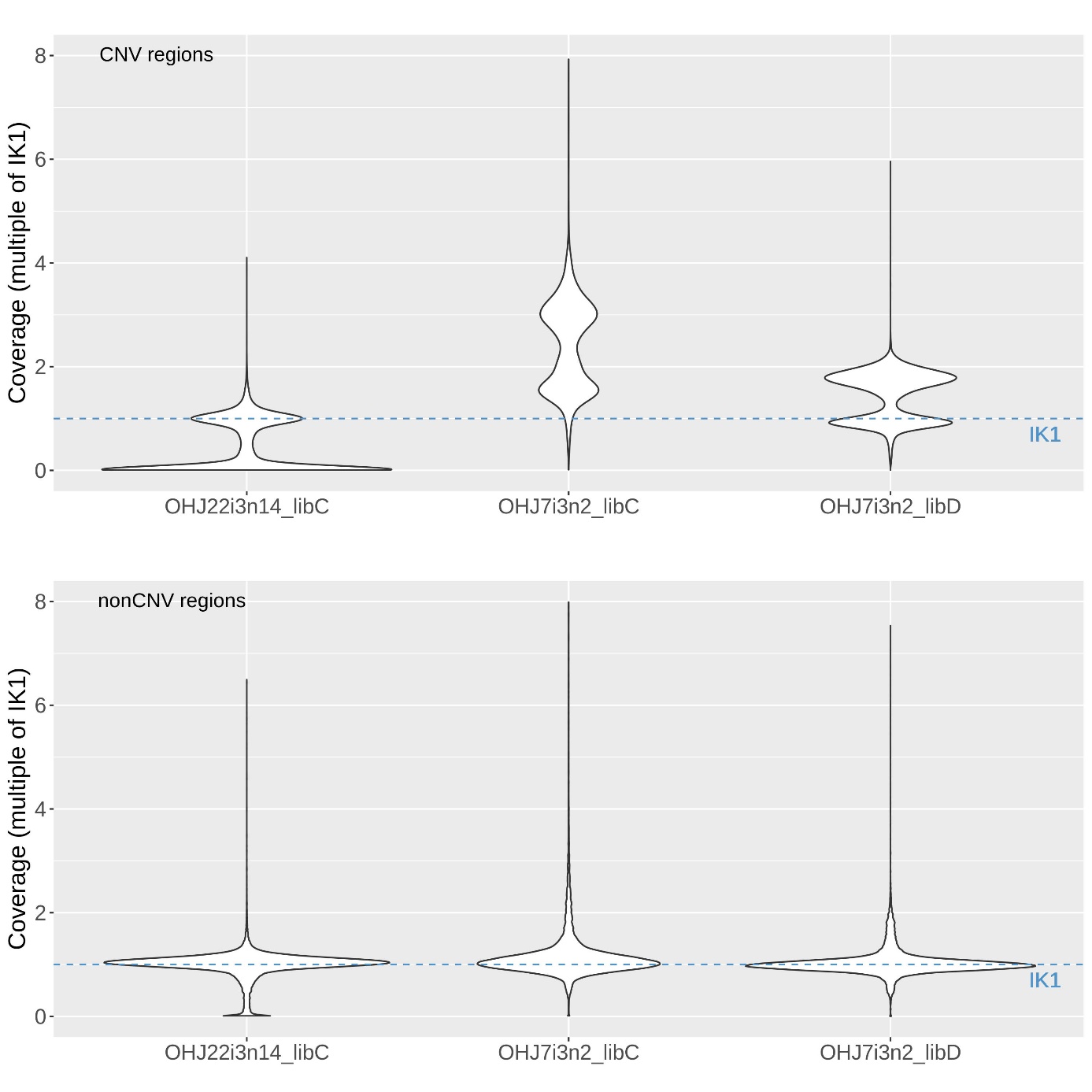


**Fig. S8: Genome-wide coverage patterns of two parental clones and their cross.** This figure summarizes per-base coverage estimates of all 5000bp windows along the genome (subdivided in CNV regions and non-CNV regions, respectively). Coverages of the two parental clones (OHJ22i3n14, OHJ7i3n2) are expressed as multiple of the coverage of the crossed offspring clone (IK1). The dashed line at 1 refers to the crossed offspring IK1. The extensions “libC” and “libD” designate co-prepared libraries as in **Tab S4**.


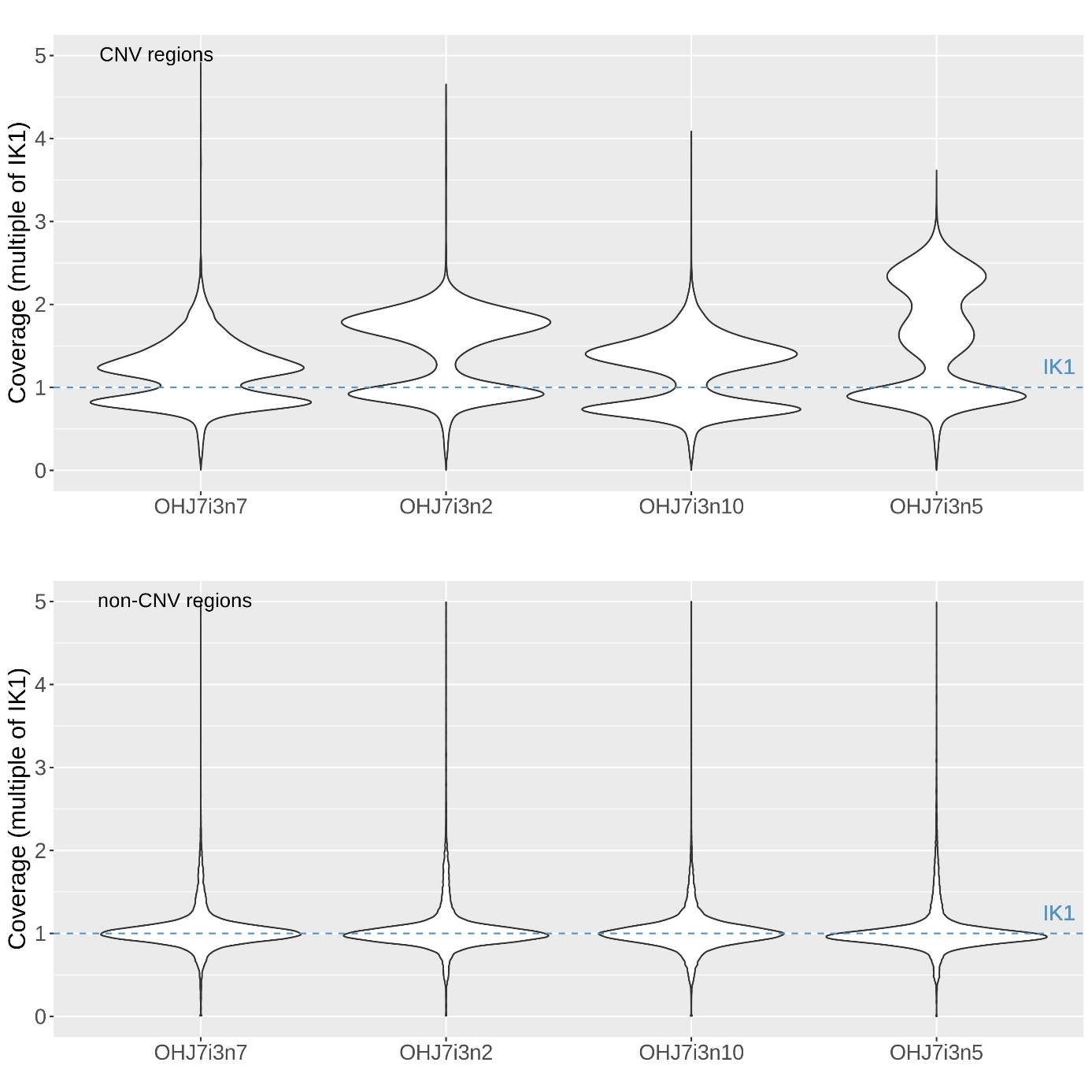


**Fig. S9: Genome-wide coverage patterns of multiple clones of a selfed line.** This figure summarizes per-base coverage estimates of all 5000bp windows along the genome (subdivided in CNV regions and non-CNV regions, respectively). Coverages of the four clones are expressed as multiple of the coverage of the crossed offspring clone (IK1). The dashed line at 1 refers to the crossed offspring IK1. Clones are ordered in ascending genome size: OHJ7i3n7 (536Mbp), OHJ7i3n2 (560MBp), OHJ7i3n10 (568Mbp), OHJ7i3n5 (644Mbp). Only the library preparations “D” Tab. S4 are displayed here, but library preparations “C” exhibited a very similar pattern (not shown).


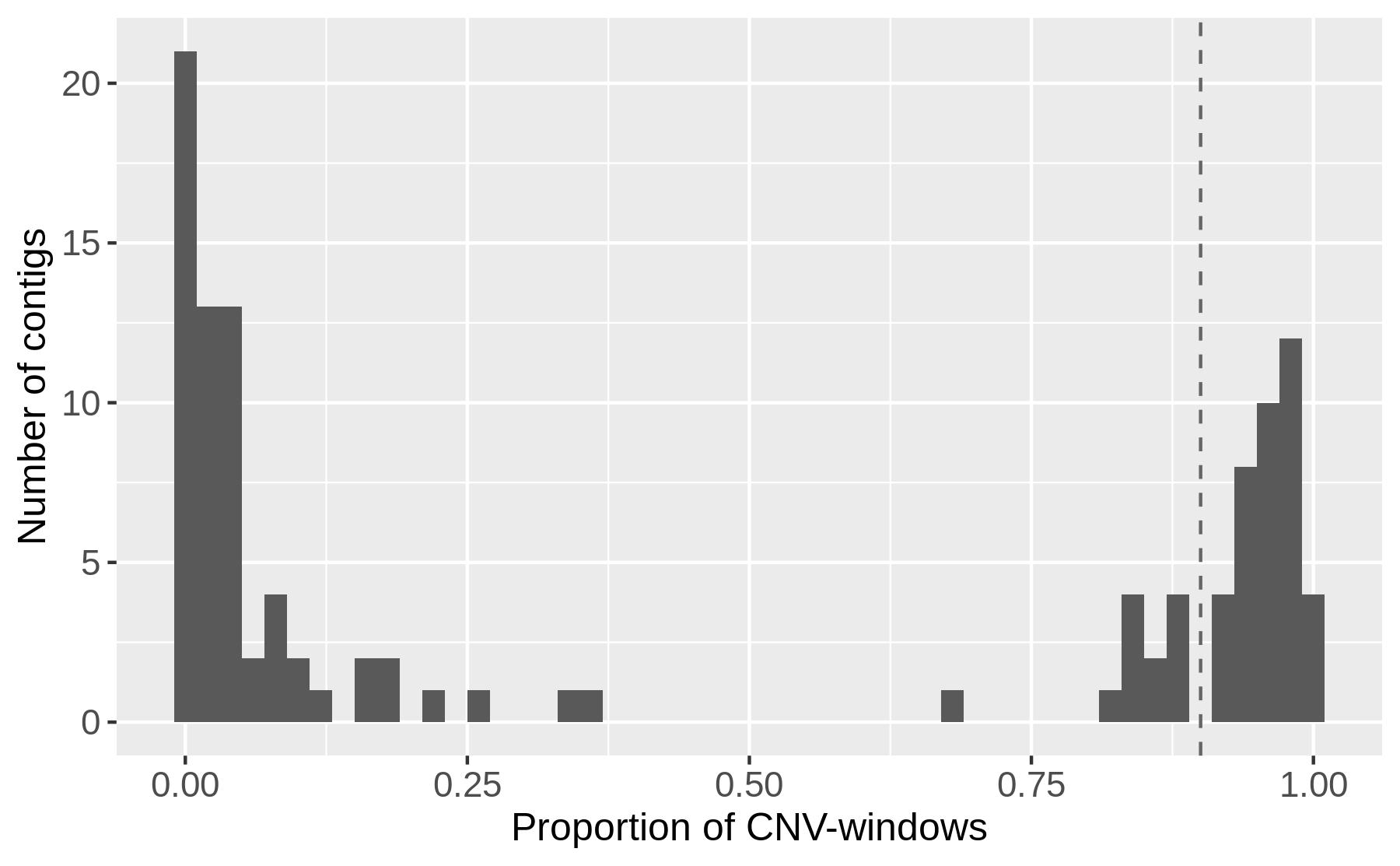


**Fig. S10: Contig space that is occupied by CNVs.** The X-axis displays the proportion of all 5kbp windows in a contig that have been classified as part of a CNV. For this graph, we only used contigs with at least 50 windows in length (i.e., contig length > 250kbp; n=114). The dashed line is our threshold at 0.9 for defining ‘B-contigs’.

**
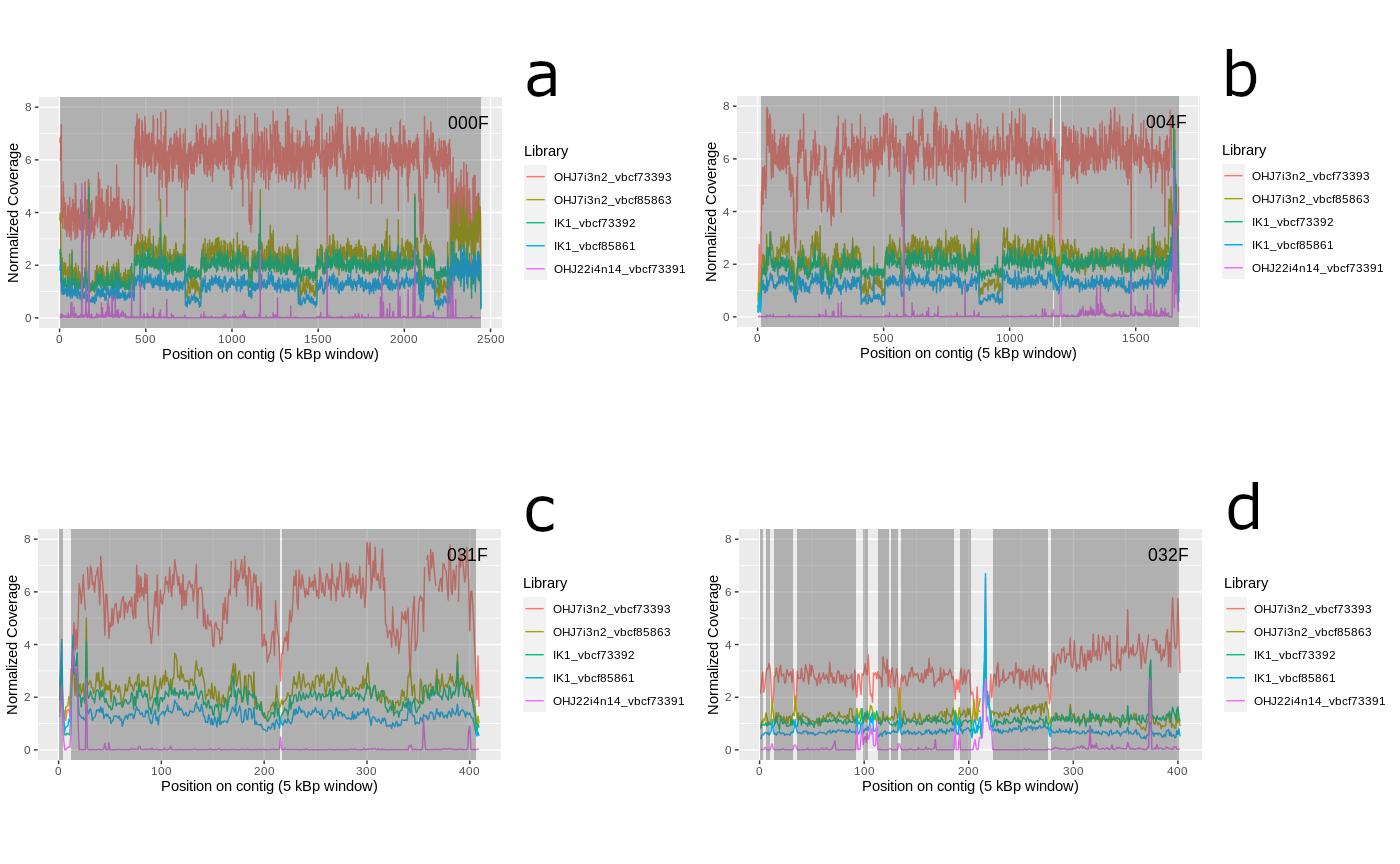
**

**Fig. S11: Examples of “B-contigs”.** B-contigs consist almost exclusively of coverage-variable regions, with a consistent coverage pattern**.** Shaded regions highlight calls of individual CNVs, while open regions are coverage breakpoints, either due to low coverage variation and/or shifts in the coverage patterns among clones. Colored lines represent normalized coverage values of different rotifer clones/libraries (with “2” equaling the coverage at exonic regions). The displayed clones are a “trio” consisting of the parents (OHJ7i3n2, 2C genome size: 560 Mbp; OHJ22i4n14, 420 Mbp) and their sexual offspring (IK1, 500 Mbp).

**
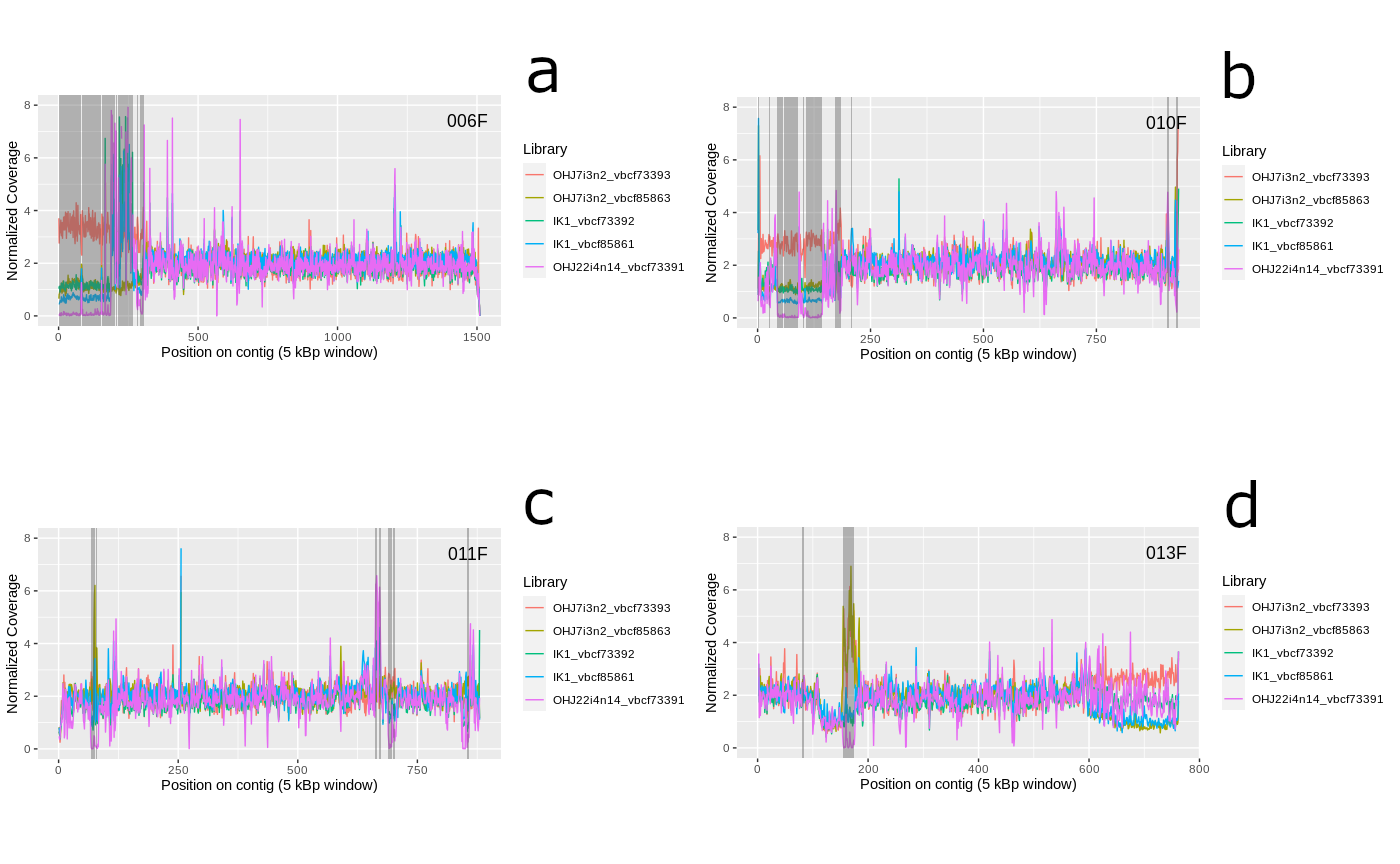
**

**Fig. S12: Example of small-scale coverage variation.** The contigs displayed here contain shorter sections that have been identified as CNVs. Shaded regions highlight calls of individual CNVs, while open regions are coverage breakpoints, either due to low coverage variation and/or shifts in the coverage patterns among clones. Colored lines represent normalized coverage values of different rotifer clones/libraries (with “2” equaling the coverage at exonic regions). The displayed clones are a “trio” consisting of the parents (OHJ7i3n2, 2C genome size: 560 Mbp; OHJ22i4n14, 420 Mbp) and their sexual offspring (IK1, 500 Mbp).


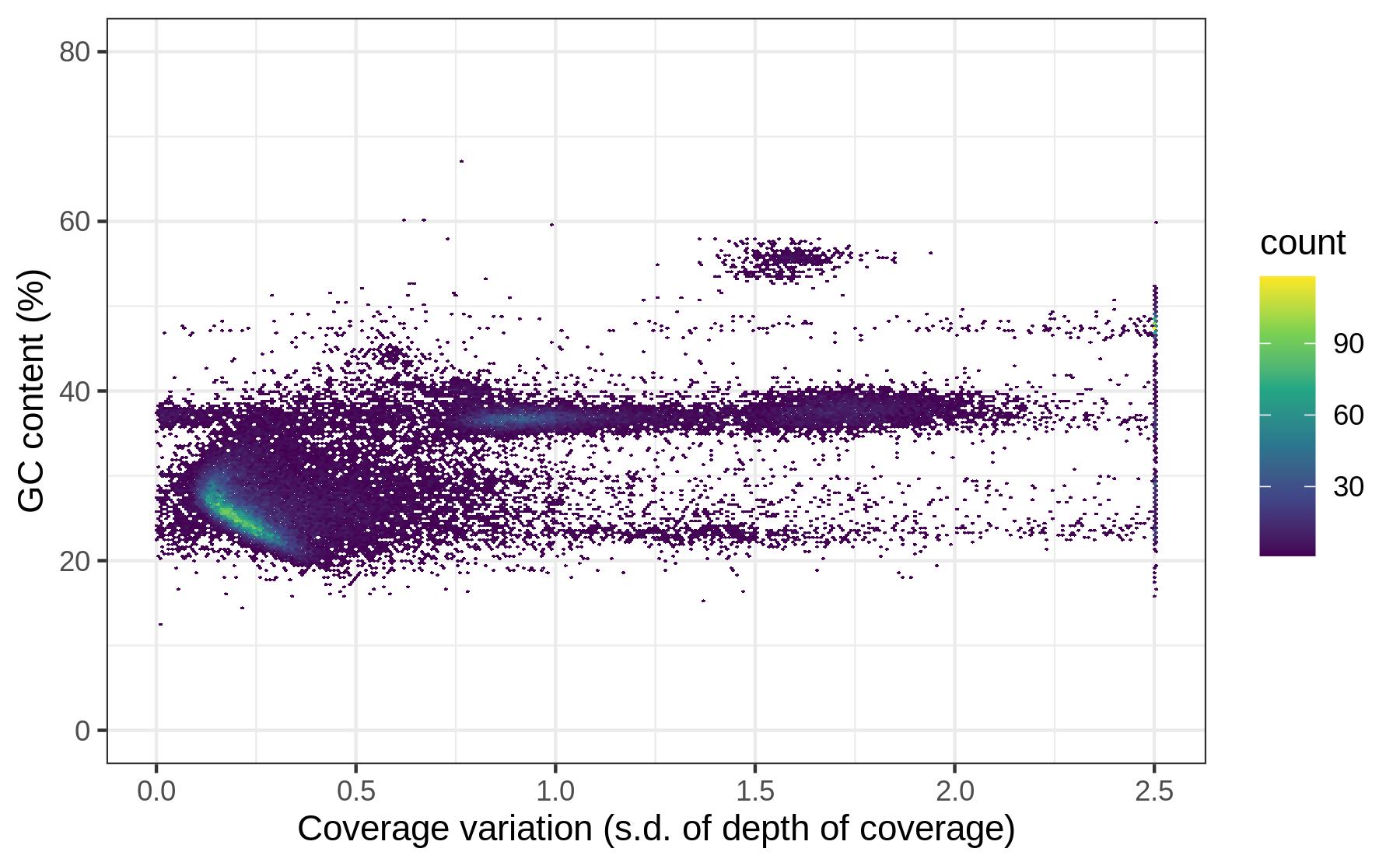


**Fig. S13: GC-content vs. coverage variability in different regions of the genome of *B. asplanchnoidis*.** Hexbin chart is based on all 5kbp windows of the assembly (bins=250, in ggplot2).


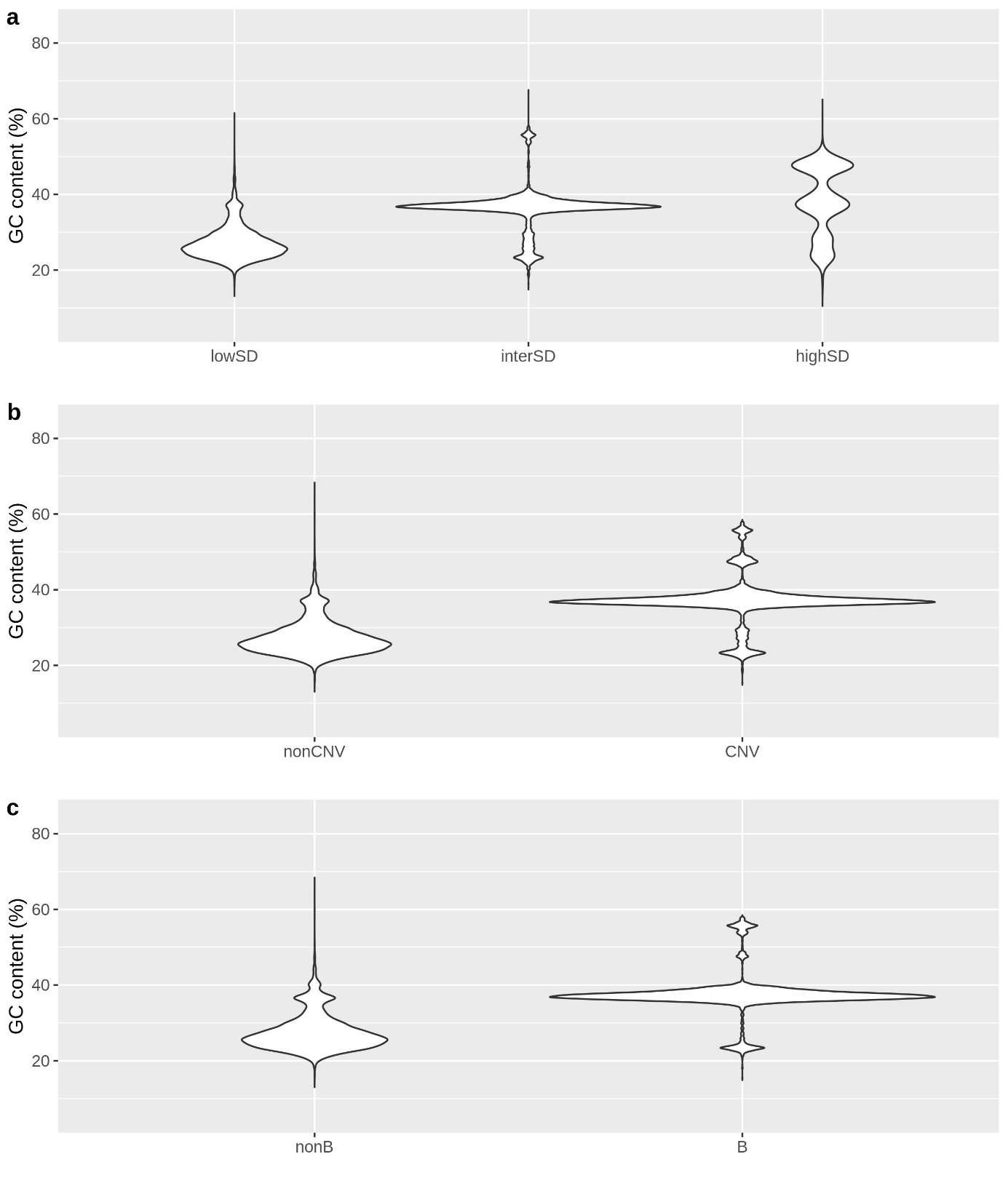


**Fig. S14: GC-content of genomic regions for in the *B. asplanchnoidis* genome.** **a** Regions defined by coverage variability, **b** CNV regions (i.e., elevated coverage variability & locally consistent coverage patterns), **c** “B-contigs” (contigs consisting of >90% CNVs). For **c**, only contigs with at least 50 windows were considered. The distributions shown here are based on GC-contents calculated from 5000-bp windows. To prevent losses in precision, the last window of a contig was excluded (since it was always shorter than 5000 bp).


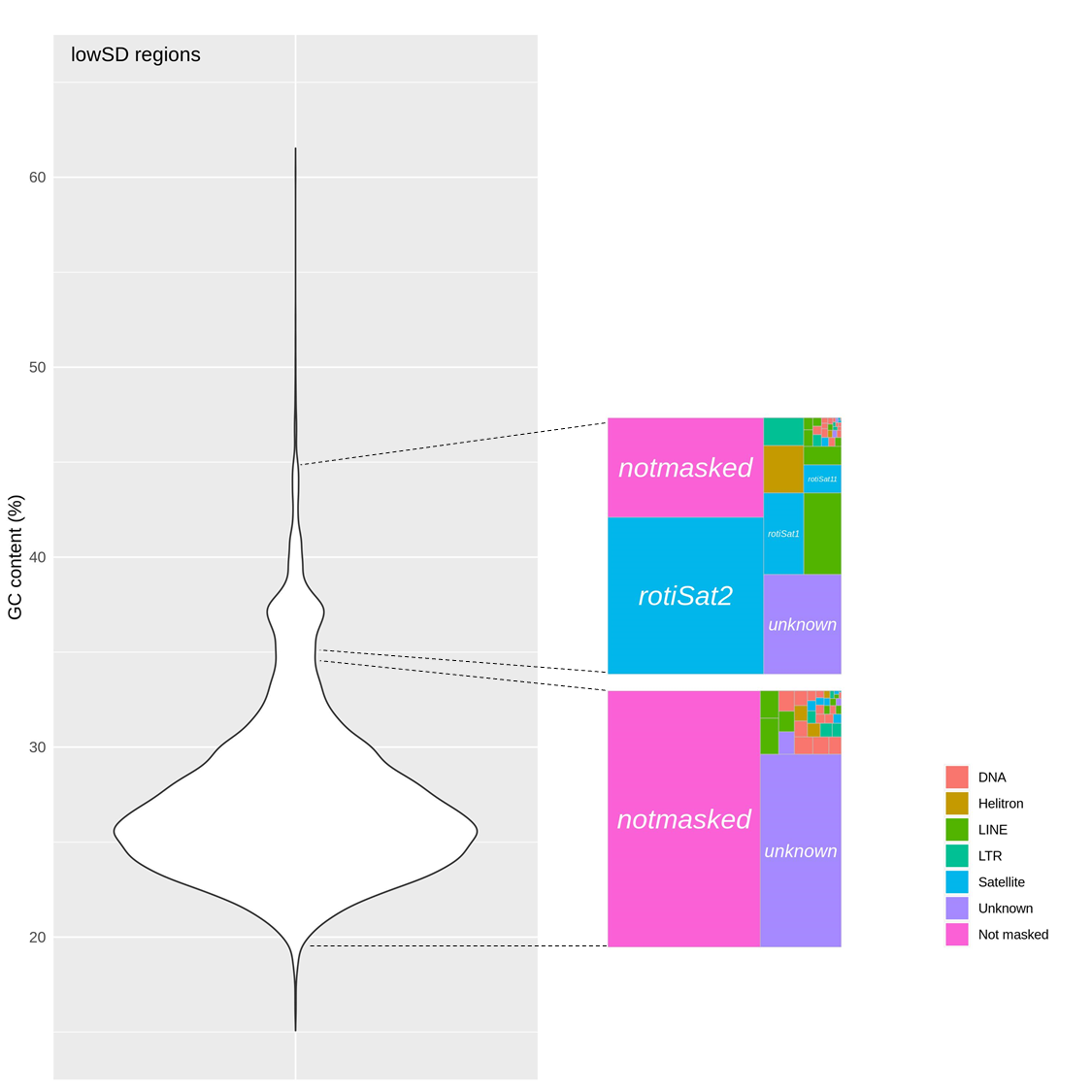


**Fig. S15: Repeat composition of various GC fractions of lowSD regions**. ‘Unknown’ refers to regions that were masked as repeats but could not be ascribed to any of the above categories. ‘Not masked’ refers to regions that were not identified as repeats by repeatModeler2.


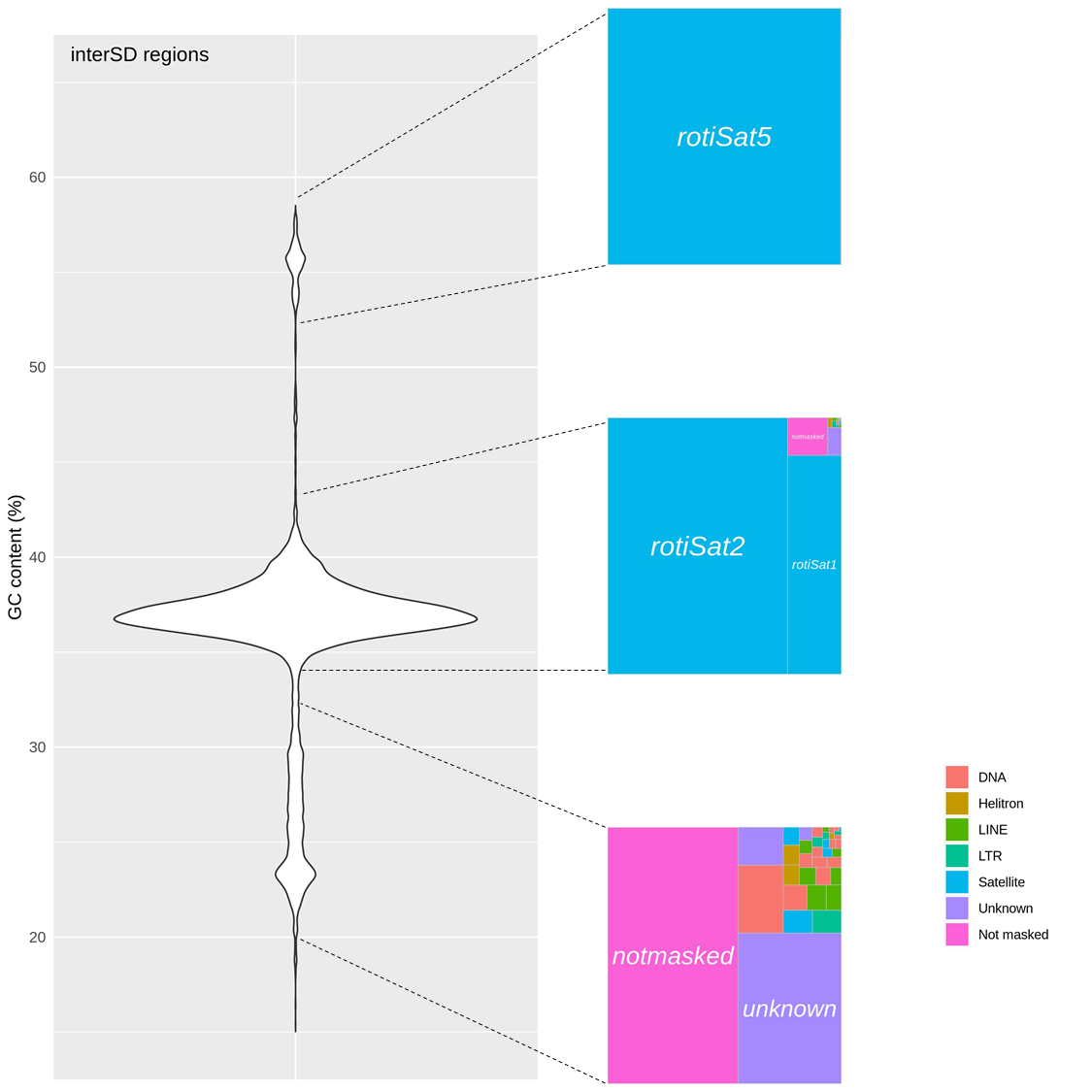


**Fig. S16: Repeat composition of various GC fractions of interSD regions.** ‘Unknown’ refers to regions that were masked as repeats but could not be ascribed to any of the above categories. ‘Not masked’ refers to regions that were not identified as repeats by repeatModeler2.


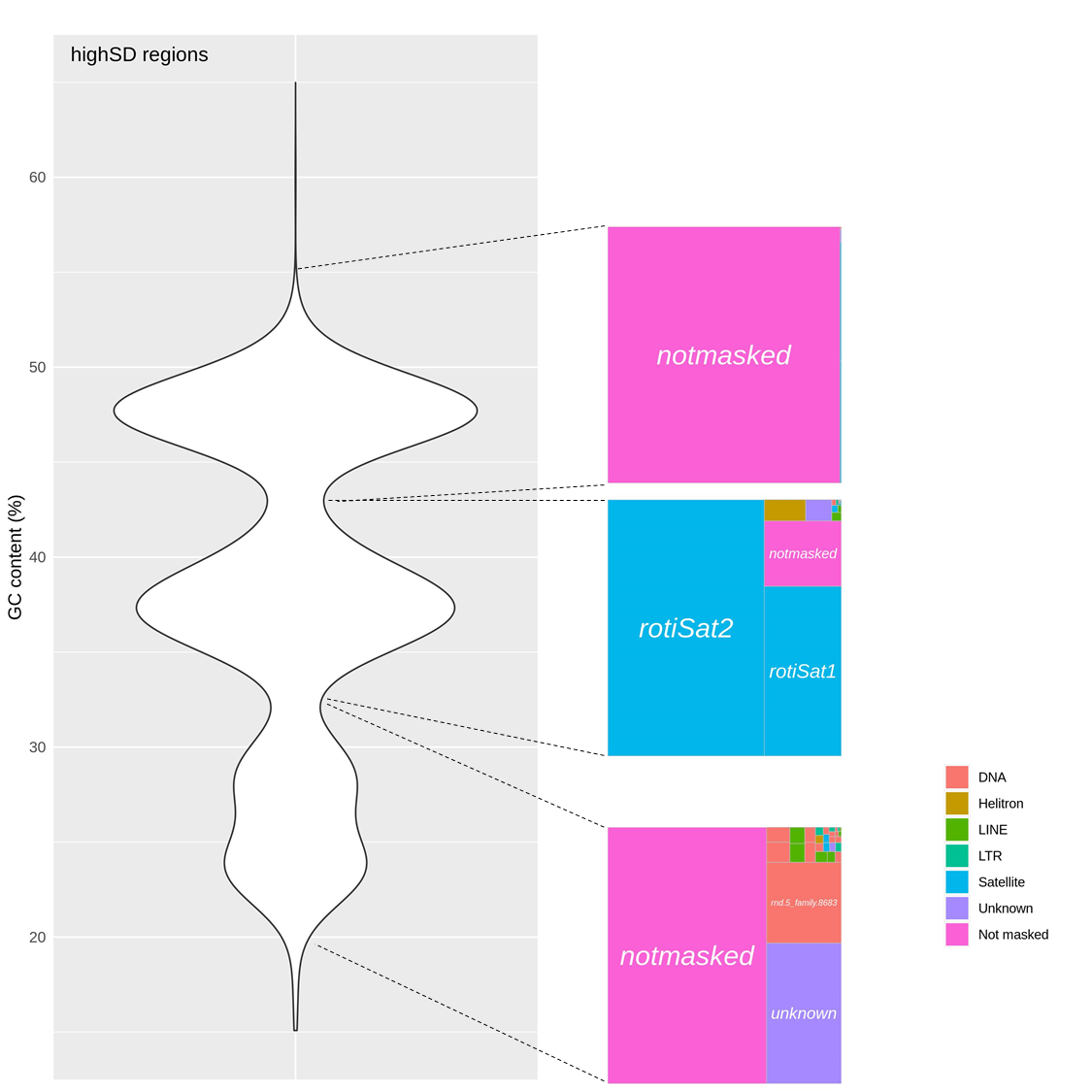


**Fig. S17: Repeat composition of various GC fractions of highSD regions.** ‘Unknown’ refers to regions that were masked as repeats but could not be ascribed to any of the above categories. ‘Not masked’ refers to regions that were not identified as repeats by repeatModeler2.


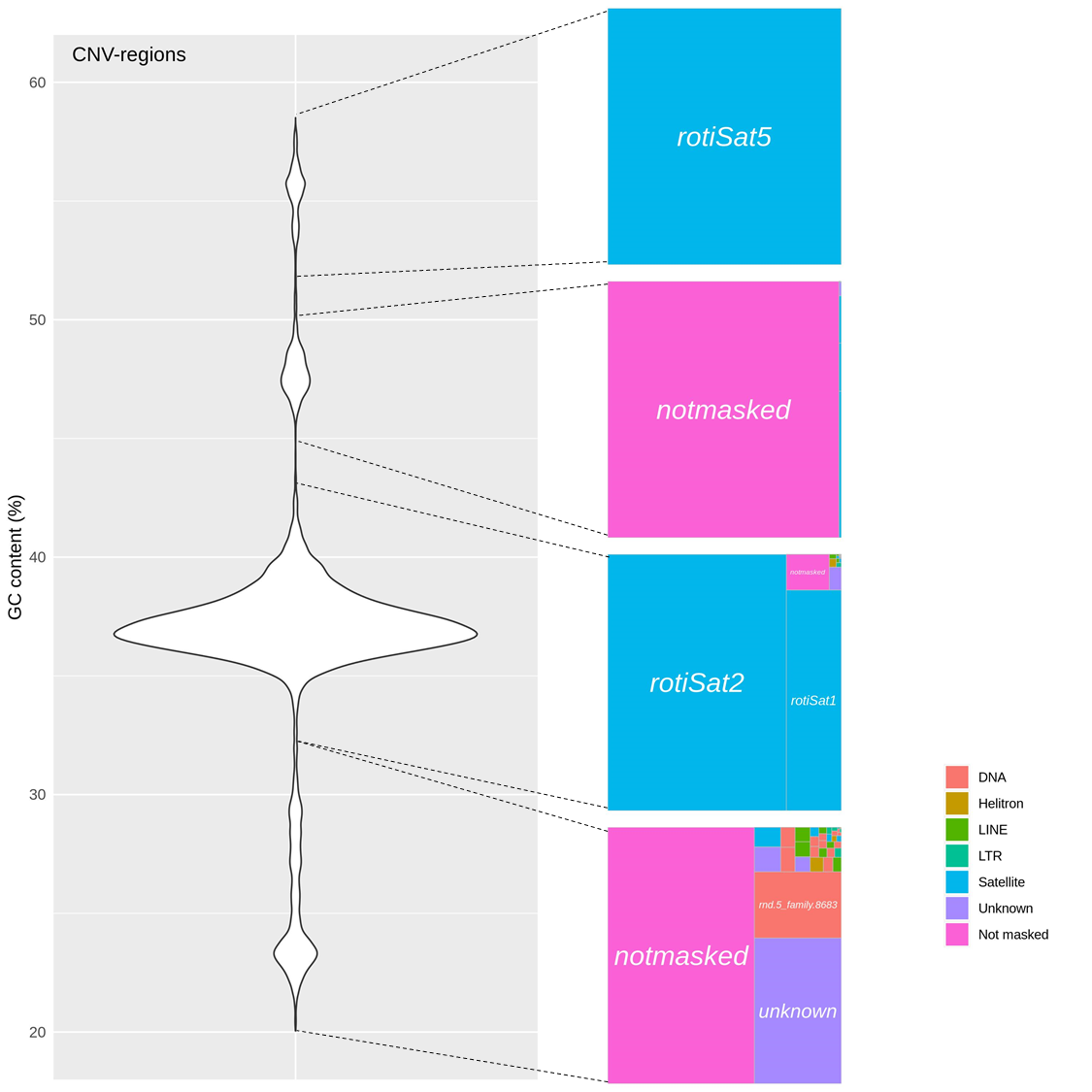


**Fig. S18: Repeat composition of various GC fractions of CNV regions.** ‘Unknown’ refers to regions that were masked as repeats but could not be ascribed to any of the above categories. ‘Not masked’ refers to regions that were not identified as repeats by repeatModeler2.


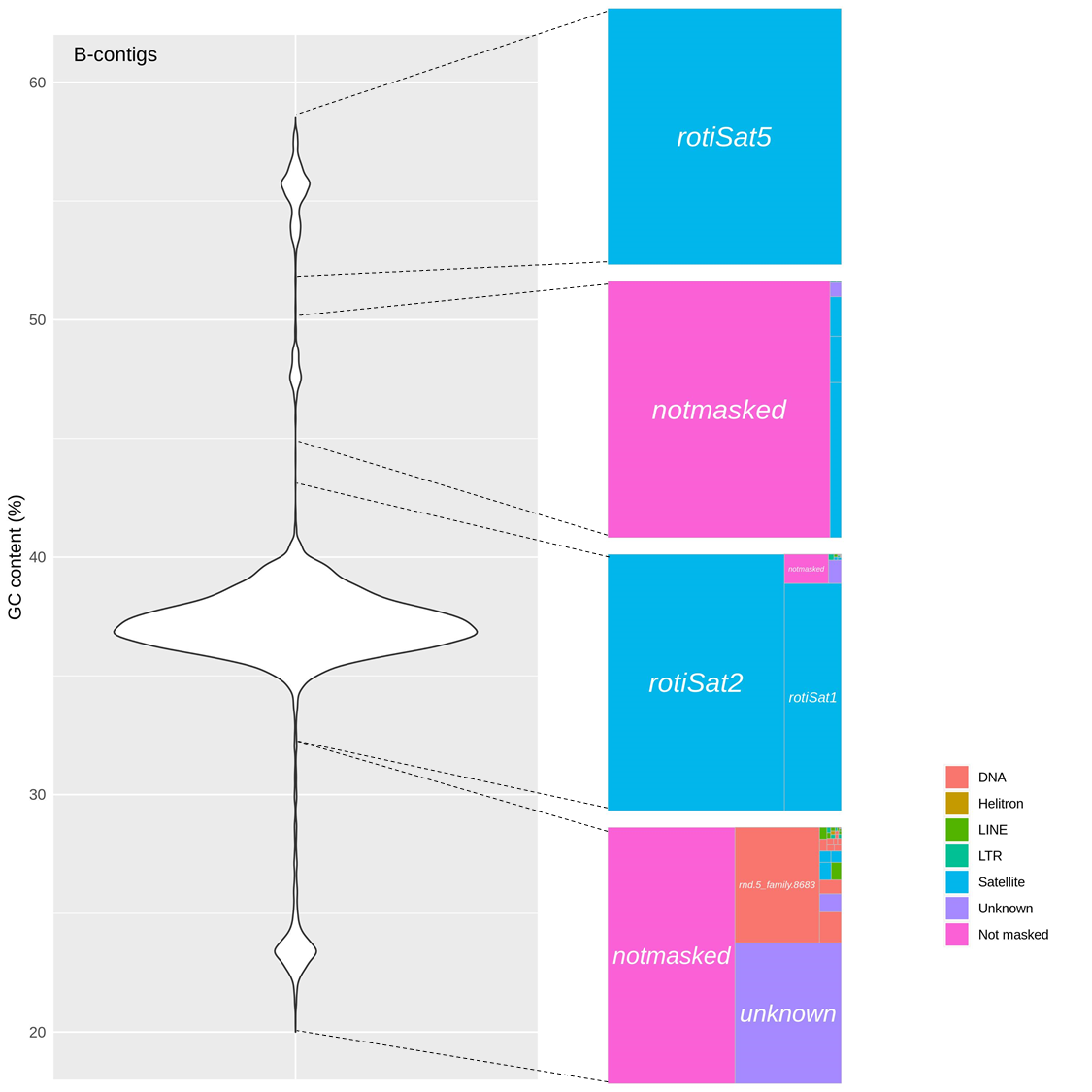


**Fig. S19: Repeat composition of various GC fractions of B-contigs.** ‘Unknown’ refers to regions that were masked as repeats but could not be ascribed to any of the above categories. ‘Not masked’ refers to regions that were not identified as repeats by repeatModeler2.

**Tab. S1: Outline of transcriptome and proteome libraries used for gene annotation.**

| Species \| clone | N  transcripts (T) \| protein seqs (P) | Reference |
| --- | --- | --- |
| *B. asplanchnoidis* \| ohj7i3n10 | T: 21157 |  |
| *B. asplanchnoidis* \| ohj22 | P: 12547 | [6] |
| *B. plicatilis* \| Tokyo1 | P: 12484 | [6] |
| *B. rotundiformis* \| Italy2 | P: 11050 | [6] |
| B. spec \| TiscarSM28 | P: 12085 | [6] |

**Table S2: BUSCO completeness statistics** of *B. asplanchniodis* genome in comparison to two previously published genomes of related *Brachionus* species.

| BUSCO (Metazoa n=978) | *B. asplanchnoidis* | *B. plicatilis* | *B. calyciflorus* |
| --- | --- | --- | --- |
| Complete % | 90.6 | 95.0 | 90.7 |
| Single complete % | 87.9 | 91.7 | 88.0 |
| Duplicated complete % | 2.7 | 3.3 | 2.7 |
| Fragmented % | 2.0 | 2.0 | 2.0 |
| Missing % | 7.4 | 3.0 | 7.3 |

Data sources: Published genome of *B. plicatilis* by [4] and of *B. calyciflorus* by [5]

**Table S3: Summary statistics of B. *asplanchnoidis* annotation** in comparison to annotations of previously published genomes from related *Brachionus* species [6]. For reasons of data availability, this comparison does not include more species of the *B. plicatilis* species complex.

|  | *B. asplanchnoidis* | *B. asplanchnoidis* ohj22 | *B. rotundiformis* Italy2 | *B.* spec  TiscarSM28 | *B. plicatilis* Tokyo1 |
| --- | --- | --- | --- | --- | --- |
| **Number** |  |  |  |  |  |
| Gene | 16667 | 12547 | 11050 | 12085 | 12484 |
| mRNA | 17419 | 12547 | 11050 | 12085 | 12484 |
| Exon | 92799 | 87600 | 91192 | 96546 | 93603 |
| CDS | 96847 | 87188 | 90882 | 96132 | 93249 |
| **Mean** |  |  |  |  |  |
| mRNAs/gene | 1.05 | 1 | 1 | 1 | 1 |
| Exons/mRNA | 5.33 | 6.98 | 8.25 | 7.99 | 7.5 |
| CDSs/mRNA | 5.56 | 6.95 | 8.22 | 7.95 | 7.47 |
| **Median length** |  |  |  |  |  |
| Gene | 1999 | 1839 | 1874 | 1912 | 1926.5 |
| mRNA | 2060 | 1839 | 1874 | 1912 | 1926.5 |
| Exon | 153 | 62 | 70 | 61 | 60 |
| Intron | 59 | 66 | 58 | 63 | 67 |
| CDS | 141 | 62 | 70 | 62 | 60 |
| **Total space** |  |  |  |  |  |
| Gene | 59373627 | 29922141 | 25565912 | 29432444 | 31741372 |
| mRNA | 59373549 | 29922141 | 25565912 | 29432444 | 31741372 |
| Exon | 28793789 | 15401203 | 16387408 | 16563187 | 16266105 |
| CDS | 27027022 | 15373829 | 16368700 | 16537800 | 16239684 |
| **Single** |  |  |  |  |  |
| Exon mRNA | 2881 | 514 | 249 | 315 | 444 |
| CDS mRNA | 2907 | 528 | 257 | 323 | 460 |

**Table S4: Information on short-read sequencing libraries**

| **Clone** | **Origin** | **GS^1^ (Mbp)** |  | **Method** | **Prep.^2^** | **max. FS^3^ (Bp)** | **avg. FS^3^ (Bp)** |
| --- | --- | --- | --- | --- | --- | --- | --- |
| OHJ82 | natural clone | 404 |  | westburg | F | 2000 | 503 |
|  |  |  |  | kapa | B | 600 | 450 |
| OHJ22 | natural clone | 412 |  | kapa | A | 600 | 450 |
|  |  |  |  | kapa | B | 600 | 450 |
| OHJ104 | natural clone | 462 |  | westburg | F | 2000 | 499 |
|  |  |  |  | kapa | B | 600 | 450 |
| OHJ97 | natural clone | 470 |  | westburg | F | 2000 | 499 |
|  |  |  |  | kapa | B | 600 | 450 |
| OHJ96 | natural clone | 492 |  | kapa | B | 600 | 450 |
| OHJ98 | natural clone | 504 |  | kapa | B | 600 | 450 |
| OHJ105 | natural clone | 520 |  | westburg | F | 2000 | 493 |
|  |  |  |  | kapa | B | 600 | 450 |
| OHJ7 | natural clone | 532 |  | westburg | E | 1000 | 386 |
|  |  |  |  | kapa | A | 600 | 450 |
|  |  |  |  | kapa | B^4^ | 600 | 450 |
|  |  |  |  | kapa | B^4^ | 600 | 450 |
| OHJ13 | natural clone | 536 |  | westburg | F | 2000 | 516 |
|  |  |  |  | kapa | B | 600 | 450 |
| OHJ22i3n14 | selfed line | 420 |  | westburg | C | 1000 | 545 |
| OHJ7i3n7 | selfed line | 536 |  | westburg | C | 1000 | 495 |
|  |  |  |  | westburg | D | 3000 | 1149 |
| OHJ7i3n2 | selfed line | 560 |  | westburg | C | 1000 | 632 |
|  |  |  |  | westburg | D | 3000 | 1228 |
| OHJ7i3n10 | selfed line | 568 |  | westburg | C | 1000 | 589 |
|  |  |  |  | westburg | D | 3000 | 1093 |
| OHJ7i3n5 | selfed line | 644 |  | westburg | C | 1000 | 496 |
|  |  |  |  | westburg | D | 3000 | 1129 |
| IK1 | selfed line cross | 500 |  | westburg | C | 1000 | 514 |
|  |  |  |  | westburg | D | 2000 | 1001 |
|  |  |  |  |  |  |  |  |
| ^1^ genome size (2C) estimated by flow cytometry in [3] | | | | | |  |  |
| ^2^ Indicates co-prepared libraries | |  |  |  |  |  |  |
| ^3^ Fragment size (FS) of the library | | |  |  |  |  |  |
| ^4^ Both derive from the same original preparation, but one was re-sequenced and had an intermediate PCR-step | | | | | | | |

**Table S5: Partial correlations^1^ between the proportion of reads in one of three GC fractions (26%, 36%, and 48% GC) and genome size**

| **GC fraction** | ***t*** | ***df*** | **Pearson's *r*** | ***P*** |
| --- | --- | --- | --- | --- |
| 26% | -3.67 | 27 | -0.58 | 0.0010 |
| 36% | 4.21 | 27 | 0.63 | 0.0003 |
| 48% | 2.48 | 27 | 0.43 | 0.0199 |

**^1^** after removing effect of library preparation.

**Table S6: Alignment statistics of short reads to reference genome.**

| **Rotifer clone** | **Library ID** | **TAR (%)** | **CCA (%)** | **CCA1 (%)** | **DAS (%)** |
| --- | --- | --- | --- | --- | --- |
| IK1 | vbcf73392 | 96.5 | 91.4 | 30.9 | 5.1 |
| IK1 | vbcf85861 | 96.1 | 94.0 | 36.5 | 2.1 |
| OHJ104 | vbcf102861 | 94.7 | 91.9 | 42.1 | 2.8 |
| OHJ105 | vbcf102863 | 95.9 | 93.0 | 34.6 | 2.9 |
| OHJ13 | vbcf102864 | 96.1 | 93.6 | 34.2 | 2.5 |
| OHJ22i4n14 | vbcf73391 | 94.9 | 90.8 | 36.7 | 4.1 |
| OHJ7 | vbcf100244 | 94.1 | 92.8 | 34.1 | 1.3 |
| OHJ7i3n10 | vbcf73394 | 98.4 | 95.8 | 25.4 | 2.6 |
| OHJ7i3n10 | vbcf85864 | 97.7 | 96.3 | 37.8 | 1.4 |
| OHJ7i3n2 | vbcf73393 | 98.4 | 96.4 | 22.8 | 2.0 |
| OHJ7i3n2 | vbcf85863 | 95.3 | 93.5 | 33.5 | 1.8 |
| OHJ7i3n5 | vbcf73395 | 97.6 | 93.1 | 26.5 | 4.5 |
| OHJ7i3n5 | vbcf85865 | 96.7 | 95.1 | 32.2 | 1.5 |
| OHJ7i3n7 | vbcf73396 | 97.5 | 93.6 | 25.9 | 3.9 |
| OHJ7i3n7 | vbcf85862 | 98.2 | 96.9 | 38.1 | 1.3 |
| OHJ82 | vbcf102860 | 94.7 | 91.8 | 43.0 | 3.0 |
| OHJ97 | vbcf102862 | 95.8 | 92.9 | 38.2 | 2.9 |
| OHJ104 | mbl2016 | 94.6 | 92.9 | 52.0 | 1.8 |
| OHJ105 | mbl2016 | 95.7 | 94.0 | 42.0 | 1.6 |
| OHJ13 | mbl2016 | 96.6 | 95.0 | 45.5 | 1.7 |
| OHJ22 | mbl2015 | 94.8 | 91.6 | 48.4 | 3.3 |
| OHJ22 | mbl2016 | 95.6 | 93.3 | 51.4 | 2.3 |
| OHJ7 | mbl2015 | 97.2 | 95.1 | 49.6 | 2.1 |
| OHJ7 | mbl2016 | 97.5 | 96.2 | 52.9 | 1.3 |
| OHJ82 | mbl2016 | 95.3 | 93.5 | 49.4 | 1.8 |
| OHJ96 | mbl2016 | 95.9 | 94.3 | 47.4 | 1.6 |
| OHJ97 | mbl2016 | 96.4 | 94.8 | 49.6 | 1.6 |
| OHJ98 | mbl2016 | 96.1 | 94.3 | 50.3 | 1.8 |
| OHJ7 | mbl2019 | 97.4 | 96.0 | 42.9 | 1.4 |
| TAR = Total alignment rate; CCA = Concordantly aligned reads; CCA1 = Concordantly aligned reads (exactly once); DAS = Discordantly aligned reads and singletons | | | | | |

**Table S7: Classification of ‘B-contigs’ in terms of the proportion of 5kbp windows that are CNV**

| **Contig** | **Total 5kbp windows** | **CNV-windows** | **Proportion** |
| --- | --- | --- | --- |
| 000F | 2444 | 2436 | 0.997 |
| 057F | 166 | 165 | 0.994 |
| 058F | 163 | 162 | 0.994 |
| 071F | 109 | 108 | 0.991 |
| 004F | 1671 | 1651 | 0.988 |
| 083F | 79 | 78 | 0.987 |
| 084F | 78 | 77 | 0.987 |
| 086F | 72 | 71 | 0.986 |
| 048F | 210 | 207 | 0.986 |
| 091F | 66 | 65 | 0.985 |
| 099F | 59 | 58 | 0.983 |
| 059F | 155 | 152 | 0.981 |
| 109F | 50 | 49 | 0.980 |
| 061F | 146 | 143 | 0.979 |
| 055F | 175 | 171 | 0.977 |
| 022F | 564 | 550 | 0.975 |
| 031F | 409 | 396 | 0.968 |
| 094F | 62 | 60 | 0.968 |
| 097F | 61 | 59 | 0.967 |
| 095F | 60 | 58 | 0.967 |
| 062F | 146 | 141 | 0.966 |
| 053F | 175 | 169 | 0.966 |
| 047F | 232 | 224 | 0.966 |
| 110F | 52 | 50 | 0.962 |
| 036F | 353 | 337 | 0.955 |
| 044F | 262 | 250 | 0.954 |
| 064F | 135 | 128 | 0.948 |
| 070F | 114 | 108 | 0.947 |
| 068F | 108 | 102 | 0.944 |
| 054F | 176 | 166 | 0.943 |
| 067F | 122 | 115 | 0.943 |
| 106F | 52 | 49 | 0.942 |
| 107F | 52 | 49 | 0.942 |
| 080F | 83 | 78 | 0.940 |
| 088F | 69 | 64 | 0.928 |
| 074F | 107 | 99 | 0.925 |
| 056F | 173 | 160 | 0.925 |
| 045F | 252 | 231 | 0.917 |

**Table S8: PCR-primers used for verification of selected CNV-loci**

| **Locus** |  | **Position in genome** | | |  | **PCR primers** | | | | | | |
| --- | --- | --- | --- | --- | --- | --- | --- | --- | --- | --- | --- | --- |
|  |  |  |  |  |  |  |  |  |  |  |  |  |
|  |  | **Contig** | **Start** | **End** |  | **Orientation** |  | **Sequence (5'->3')** | **Length (mer)** | **GC (%)** | **Tm (°C)** | **Amplicon (bp)** |
|  |  |  |  |  |  |  |  |  |  |  |  |  |
|  |  |  |  |  |  |  |  |  |  |  |  |  |
| **TA_000F** |  | 000F | 471 | 681 |  | forward |  | TCCAAAGGACGTTGATTAACCTG | 23 | 43 | 58.9 | 210 |
|  |  |  |  |  |  | reverse |  | GGTTGATCAAGCTCCTTAGGAC | 22 | 50 | 60.3 |  |
|  |  |  |  |  |  |  |  |  |  |  |  |  |
| **TA_003F** |  | 003F | 3642291 | 3642472 |  | forward |  | TTTCGGGTAAGTCGACTTATTCC | 23 | 43 | 58.9 | 181 |
|  |  |  |  |  |  | reverse |  | ATCCGGCATGTAGTATGAAACTG | 23 | 43 | 58.9 |  |
|  |  |  |  |  |  |  |  |  |  |  |  |  |
| **TA_001F** |  | 001F | 841341 | 841438 |  | forward |  | GCGGTCCAAATTCCCAAATTC | 21 | 47 | 57.9 | 97 |
|  |  |  |  |  |  | reverse |  | GCGTCGCAATGTTGGAAAGC | 20 | 55 | 59.4 |  |
|  |  |  |  |  |  |  |  |  |  |  |  |  |
| **TA_032F** |  | 032F | 62 | 163 |  | forward |  | GCTCATCTCTGATGGTATTTGTCC | 24 | 45 | 61.0 | 101 |
|  |  |  |  |  |  | reverse |  | CAAGGCAAAGTCATCAGTGAAGTC | 24 | 45 | 61.0 |  |

**Table S9: Data from ddPCR-measurements.**

|  | | **Estimated copies/nl** | | | **Droplet counts** | | |
| --- | --- | --- | --- | --- | --- | --- | --- |
| **Clone** | **Primer** | **Estimate** | **ConfMax** | **ConfMin** | **Positives** | **Negatives** | **Accepted** |
| ik1 | TA_001F | 40.1 | 43.6 | 38.3 | 497 | 14340 | 14837 |
| ik1 | TA_003F | 44.1 | 47.8 | 42.1 | 523 | 13708 | 14231 |
| ik1 | TA_032F | 63.4 | 67.9 | 61.0 | 751 | 13574 | 14325 |
| ik1 | TA_000F | 0.6 | 1.1 | 0.4 | 7 | 14308 | 14315 |
| neg | TA_001F | 0.0 | 0.2 | 0.0 | 0 | 15814 | 15814 |
| neg | TA_003F | 0.0 | 0.2 | 0.0 | 0 | 14881 | 14881 |
| neg | TA_032F | 0.1 | 0.4 | 0.0 | 1 | 15833 | 15834 |
| neg | TA_000F | 1.2 | 2.0 | 1.0 | 16 | 15155 | 15171 |
| ohj104 | TA_001F | 80.1 | 85.0 | 77.7 | 1072 | 15206 | 16278 |
| ohj104 | TA_003F | 58.2 | 62.6 | 56.0 | 700 | 13791 | 14491 |
| ohj104 | TA_032F | 0.2 | 0.5 | 0.1 | 2 | 15859 | 15861 |
| ohj104 | TA_000F | 0.5 | 1.0 | 0.4 | 7 | 15476 | 15483 |
| ohj105 | TA_001F | 59.7 | 64.0 | 57.4 | 715 | 13746 | 14461 |
| ohj105 | TA_003F | 45.5 | 49.8 | 43.4 | 444 | 11256 | 11700 |
| ohj105 | TA_032F | 1.3 | 2.0 | 1.0 | 16 | 14976 | 14992 |
| ohj105 | TA_000F | 1.3 | 2.0 | 1.0 | 16 | 14668 | 14684 |
| ohj13 | TA_001F | 53.2 | 57.3 | 51.1 | 658 | 14229 | 14887 |
| ohj13 | TA_003F | 48.2 | 52.1 | 46.3 | 602 | 14382 | 14984 |
| ohj13 | TA_032F | 44.9 | 48.6 | 43.0 | 552 | 14195 | 14747 |
| ohj13 | TA_000F | 0.8 | 1.3 | 0.5 | 10 | 15477 | 15487 |
| ohj22 | TA_001F | 89.6 | 94.8 | 87.0 | 1160 | 14650 | 15810 |
| ohj22 | TA_003F | 72.0 | 76.8 | 69.6 | 865 | 13705 | 14570 |
| ohj22 | TA_032F | 0.0 | 0.2 | 0.0 | 0 | 15068 | 15068 |
| ohj22 | TA_000F | 0.6 | 1.1 | 0.4 | 7 | 14428 | 14435 |
| ohj7 | TA_001F | 61.0 | 65.4 | 58.7 | 726 | 13645 | 14371 |
| ohj7 | TA_003F | 50.0 | 54.0 | 48.0 | 608 | 13992 | 14600 |
| ohj7 | TA_032F | 109.9 | 115.5 | 107.0 | 1463 | 14942 | 16405 |
| ohj7 | TA_000F | 1.3 | 2.0 | 1.0 | 17 | 15772 | 15789 |
| ohj7i3n10 | TA_001F | 68.5 | 73.2 | 66.1 | 815 | 13589 | 14404 |
| ohj7i3n10 | TA_003F | 56.7 | 61.1 | 54.4 | 636 | 12888 | 13524 |
| ohj7i3n10 | TA_032F | 133.4 | 139.9 | 130.1 | 1629 | 13570 | 15199 |
| ohj7i3n10 | TA_000F | 0.6 | 1.1 | 0.4 | 8 | 15982 | 15990 |
| ohj7i3n2 | TA_001F | 41.0 | 44.5 | 39.2 | 539 | 15200 | 15739 |
| ohj7i3n2 | TA_003F | 38.9 | 42.4 | 37.2 | 502 | 14917 | 15419 |
| ohj7i3n2 | TA_032F | 127.6 | 133.7 | 124.5 | 1680 | 14664 | 16344 |
| ohj7i3n2 | TA_000F | 0.8 | 1.3 | 0.5 | 10 | 15523 | 15533 |
| ohj7i3n5 | TA_001F | 46.2 | 50.1 | 44.2 | 529 | 13218 | 13747 |
| ohj7i3n5 | TA_003F | 40.7 | 44.5 | 38.7 | 430 | 12222 | 12652 |
| ohj7i3n5 | TA_032F | 126.7 | 133.3 | 123.4 | 1429 | 12565 | 13994 |
| ohj7i3n5 | TA_000F | 0.4 | 0.9 | 0.3 | 5 | 13668 | 13673 |
| ohj7i3n7 | TA_001F | 65.1 | 70.9 | 62.1 | 481 | 8458 | 8939 |
| ohj7i3n7 | TA_003F | 46.5 | 50.3 | 44.6 | 600 | 14874 | 15474 |
| ohj7i3n7 | TA_032F | 156.0 | 163.0 | 152.0 | 1919 | 13578 | 15497 |
| ohj7i3n7 | TA_000F | 0.5 | 1.0 | 0.3 | 7 | 16221 | 16228 |
| ohj82 | TA_001F | 74.5 | 79.2 | 72.1 | 949 | 14519 | 15468 |
| ohj82 | TA_003F | 65.1 | 69.8 | 62.7 | 727 | 12783 | 13510 |
| ohj82 | TA_032F | 0.1 | 0.4 | 0.0 | 1 | 16175 | 16176 |
| ohj82 | TA_000F | 1.1 | 1.8 | 0.8 | 14 | 14950 | 14964 |
| ohj96 | TA_001F | 57.0 | 61.3 | 54.8 | 688 | 13859 | 14547 |
| ohj96 | TA_003F | 45.7 | 49.4 | 43.8 | 590 | 14901 | 15491 |
| ohj96 | TA_032F | 54.2 | 58.3 | 52.1 | 653 | 13858 | 14511 |
| ohj96 | TA_000F | 0.3 | 0.8 | 0.2 | 4 | 14106 | 14110 |
| ohj97 | TA_001F | 62.0 | 66.1 | 59.9 | 857 | 15846 | 16703 |
| ohj97 | TA_003F | 44.5 | 48.0 | 42.7 | 620 | 16079 | 16699 |
| ohj97 | TA_032F | 55.0 | 58.9 | 53.0 | 749 | 15652 | 16401 |
| ohj97 | TA_000F | 0.3 | 0.7 | 0.2 | 4 | 15757 | 15761 |
| ohj98 | TA_001F | 57.4 | 61.7 | 55.2 | 689 | 13772 | 14461 |
| ohj98 | TA_003F | 51.1 | 55.2 | 49.1 | 604 | 13594 | 14198 |
| ohj98 | TA_032F | 1.2 | 1.9 | 0.9 | 15 | 15193 | 15208 |
| ohj98 | TA_000F | 1.3 | 2.0 | 1.0 | 16 | 15009 | 15025 |

**Table S10: GO enrichment analysis of genes that derived from a duplication event.**

| **GO.ID** | **Term** | **Annotated** | **Significant** | **Expected** | **Fisher** *P* |
| --- | --- | --- | --- | --- | --- |
| GO:0015074 | DNA integration | 274 | 212 | 74.17 | < 1e-30 |
| GO:0032264 | IMP salvage | 6 | 6 | 1.62 | 0.0004 |
| GO:0006430 | lysyl-tRNA aminoacylation | 6 | 6 | 1.62 | 0.0004 |
| GO:0006809 | nitric oxide biosynthetic process | 10 | 8 | 2.71 | 0.0007 |
| GO:0042157 | lipoprotein metabolic process | 43 | 19 | 11.64 | 0.0021 |
| GO:0007064 | mitotic sister chromatid cohesion | 9 | 7 | 2.44 | 0.0022 |
| GO:0016567 | protein ubiquitination | 57 | 26 | 15.43 | 0.0039 |
| GO:0006281 | DNA repair | 137 | 45 | 37.08 | 0.0041 |
| GO:0051014 | actin filament severing | 4 | 4 | 1.08 | 0.0054 |
| GO:0030148 | sphingolipid biosynthetic process | 6 | 5 | 1.62 | 0.0067 |
| GO:0043161 | proteasome-mediated ubiquitin ... | 47 | 21 | 12.72 | 0.0069 |
| GO:0006030 | chitin metabolic process | 92 | 38 | 24.9 | 0.0139 |
| GO:0005975 | carbohydrate metabolic process | 244 | 89 | 66.05 | 0.0145 |
| GO:0016998 | cell wall macromolecule ... | 9 | 6 | 2.44 | 0.0150 |
| GO:0006032 | chitin catabolic process | 9 | 6 | 2.44 | 0.0150 |
| GO:0006869 | lipid transport | 54 | 20 | 14.62 | 0.0166 |
| GO:0032784 | regulation of DNA-templated ... | 10 | 7 | 2.71 | 0.0197 |
| GO:0006525 | arginine metabolic process | 6 | 5 | 1.62 | 0.0198 |
| GO:0090630 | activation of GTPase activity | 3 | 3 | 0.81 | 0.0198 |
| GO:0006937 | regulation of muscle contraction | 3 | 3 | 0.81 | 0.0198 |
| GO:2000574 | regulation of microtubule motor activity | 3 | 3 | 0.81 | 0.0198 |
| GO:0006542 | glutamine biosynthetic process | 5 | 4 | 1.35 | 0.0210 |
| GO:0051016 | barbed-end actin filament capping | 5 | 4 | 1.35 | 0.0210 |
| GO:0048015 | phosphatidylinositol-mediated signaling | 12 | 7 | 3.25 | 0.0221 |
| GO:0023052 | signaling | 606 | 115 | 164.03 | 0.0239 |
| GO:0007020 | microtubule nucleation | 10 | 6 | 2.71 | 0.0290 |
| GO:0016311 | dephosphorylation | 88 | 25 | 23.82 | 0.0303 |
| GO:0051260 | protein homooligomerization | 35 | 16 | 9.47 | 0.0402 |

**Table S11: GO enrichment analysis of genes derived from a duplication event and found within CNV regions**

| **GO.ID** | **Term** | **Annotated** | **Significant** | **Expected** | **Fisher** *P* |
| --- | --- | --- | --- | --- | --- |
| GO:0007165 | signal transduction | 591 | 14 | 9.54 | 0.0007 |
| GO:0048015 | phosphatidylinositol-mediated signaling | 12 | 3 | 0.19 | 0.0008 |
| GO:0046854 | phosphatidylinositol phosphorylation | 14 | 3 | 0.23 | 0.0013 |
| GO:0008285 | negative regulation of cell proliferatio... | 5 | 2 | 0.08 | 0.0025 |
| GO:0042147 | retrograde transport endosome to Golgi | 10 | 2 | 0.16 | 0.0107 |
| GO:0016573 | histone acetylation | 14 | 2 | 0.23 | 0.0207 |
| GO:0005991 | trehalose metabolic process | 20 | 2 | 0.32 | 0.0406 |
| GO:0007017 | microtubule-based process | 191 | 11 | 3.08 | 0.0462 |
| GO:0098535 | de novo centriole assembly ... | 3 | 1 | 0.05 | 0.0477 |
| GO:2000574 | regulation of microtubule motor activity | 3 | 1 | 0.05 | 0.0477 |
| GO:0007040 | lysosome organization | 3 | 1 | 0.05 | 0.0477 |

**References for SI-citations**

1. Andrews S. FastQC: a quality control tool for high throughput sequence data. 2010.

2. Sun H, Ding J, Piednoël M, Schneeberger K. findGSE: estimating genome size variation within human and Arabidopsis using k-mer frequencies. Bioinformatics. 2017;34(4):550-7. doi: 10.1093/bioinformatics/btx637.

3. Stelzer CP, Pichler M, Stadler P, Hatheuer A, Riss S. Within-Population Genome Size Variation is Mediated by Multiple Genomic Elements That Segregate Independently during Meiosis. Genome Biol Evol. 2019;11(12):3424-35. PubMed PMID: WOS:000514900600009.

4. Han J, Park JC, Choi B-S, Kim M-S, Kim H-S, Hagiwara A, et al. The genome of the marine monogonont rotifer Brachionus plicatilis: Genome-wide expression profiles of 28 cytochrome P450 genes in response to chlorpyrifos and 2-ethyl-phenanthrene. Aquatic Toxicology. 2019;214:105230. doi: <https://doi.org/10.1016/j.aquatox.2019.105230>.

5. Kim H-S, Lee B-Y, Han J, Jeong C-B, Hwang D-S, Lee M-C, et al. The genome of the freshwater monogonont rotifer Brachionus calyciflorus. Molecular Ecology Resources. 2018;18(3):646-55. doi: 10.1111/1755-0998.12768.

6. Blommaert J, Riss S, Hecox-Lea B, Mark Welch DB, Stelzer CP. Small, but surprisingly repetitive genomes: Transposon expansion and not polyploidy has driven a doubling in genome size in a metazoan species complex. BMC Genomics. 2019;20(466). doi: <https://doi.org/10.1186/s12864-019-5859-y>.
