# Supplementary material for: Comparative analysis reveals within-population genome size variation in a rotifer is driven by large genomic elements with highly abundant satellite DNA repeat elements
Source: BMC Biol. 2021 Sep 16;19:206. doi: 10.1186/s12915-021-01134-w (PMC8447722; doi:10.1186/s12915-021-01134-w)
Supplement: Supplementary file 2 — Additional file 2. Report on genome assembly and contaminant filtering provided by VBCF (Vienna Biocenter Core Facility). [file 12915_2021_1134_MOESM2_ESM.pdf]

Whole genome sequencing study for assembly of *Brachionus asplanchnoidis* Claus-Peter Stelzer

Bence Galik
bence.galik@vbcf.ac.at
BioComp
Vienna Biocenter Core Facilities
https://www.vbcf.ac.at/facilities/bioinformatics-and-scientific-computing/

November 5, 2018

Content

1 Analysis workflow 3

2 1st genome assembly 4

3 Quality assessment 4

4 Filter contaminations 6

5 2nd genome assembly 8

6 Filter contaminations 8

7 Quality assessment 8

8 Summary of the result files 11

References 12

1 Analysis workflow

The aim of this whole genome sequencing study was to create a draft assembly for *Brachionus asplanchnoidis*. The sample was sequenced with PacBio Sequel instrument. First, only one SMRTcell was used to generated sequencing data in order to look for bacterial contamination and filter it. Later based on the result from the first analysis a second SMRTcell was used to create the necessary amount of data for the final genome assembly.

Please make sure that you have downloaded the raw results from the NGS facility, as we do not store the unaligned files.

First the raw reads were assembled applying HGAP4 pipeline [1]. Next the quality of the genome assembly was evaluated using QAST v4.4 [2]. The contamination was checked using CLARK [3] against all available bacterial genomes from NCBI.

The statistics about the reads from the first and second SMRTcells are represented in Table 1. The results of the quality assessment are presented in later sections.

**(A)**	
Number of Subread Bases	8,317,249,981
Number of Subreads	403,580
Subread Length N50	36,256 bp
Subread Length Mean	20,608.68 bp

**(B)**	
Number of Subread Bases	8,009,699,898
Number of Subreads	510,064
Subread Length N50	29,884 bp
Subread Length Mean	15,703.32 bp

Table 1. (A) Summary of read statistics from the first test assembly. (B) Summary of read statistics from the final genome assembly.

2 First genome assembly

The genome was assembled into 32 contigs using HGAP4 pipeline with default parameters.

The reason of the low number is that the input dataset from one SMRTcell did not have enough coverage to assemble the genome properly.

3 Quality assessment

The quality assessment was done using QUAST v4.4. Table 2 contains the main metrics of the assembly and Figure 1 shows the cumulative lengths of the contigs.

You can find additional files in the *Results* folder.

Numer of contigs (>= 0 bp)	32
Total length (>= 0 bp)	850,930
Total length (>= 25000 bp)	677,157
Total length (>= 50000 bp)	384,470
Total number of contigs	32
Largest contig	133,877
Total length	850,930
GC (%)	30.03
N50	49,772
N75	43,672
L50	5
L75	10
Numer of N's per 100 kbp	0.00

Table 2. Summary of the assembly metrics.

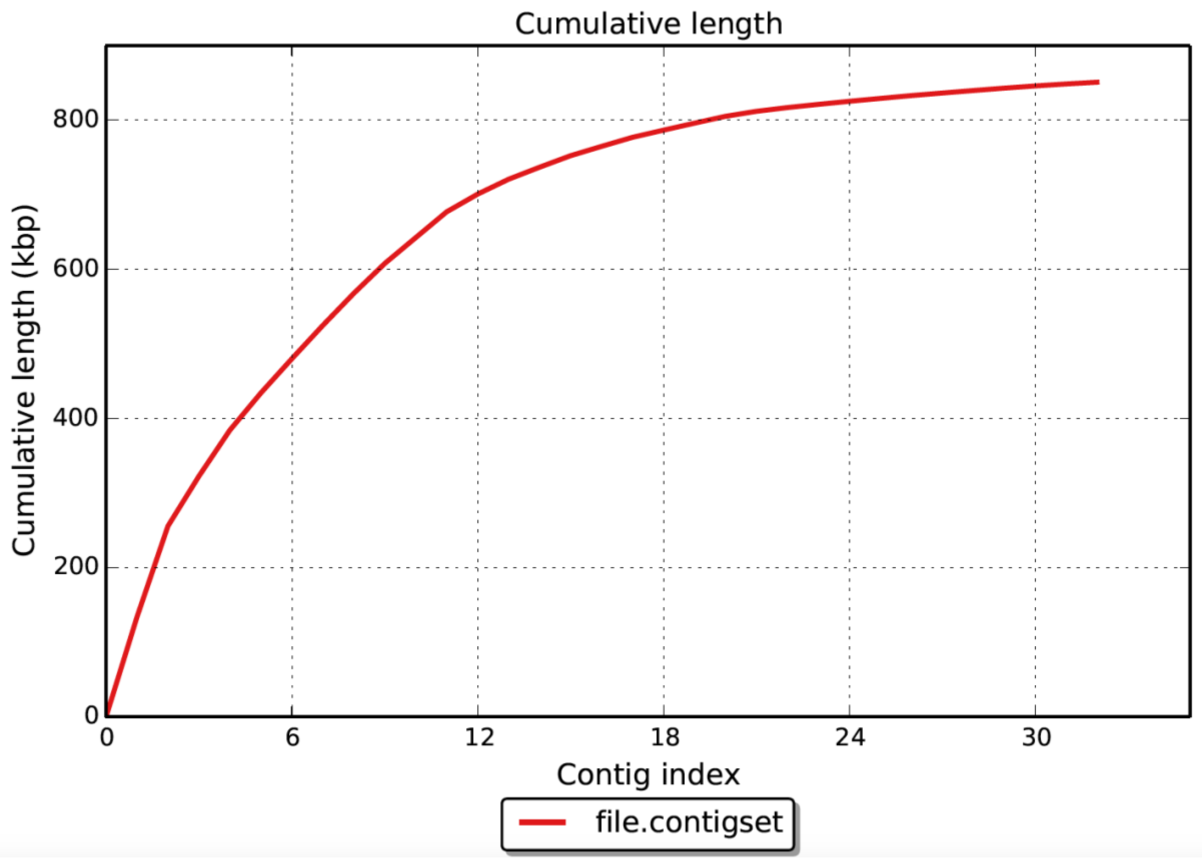

Figure 1. Cumulative length plot of contigs.

4 Filter contaminations

According to customer the *Brachionus asplanchnoidis* have some symbiotic bacteria but the we did not have any information about the exact species.

Therefore, in order to check any possibility for bacterial contamination the assembled contigs were analyzed using CLARK. The tool can assign a taxonomic group to a contig/read based on the k-mer content of the query/object. The reference genomes were downloaded from NCBI.

The Gamma value (> 0.1) was used to filter the results. The Gamma values shows the ratio between the total number of kmer hits found in the object against all targets and the number of k-mers in the object.

Also, the contigs were check using BLAST but no long (bacterial) significant hits were found.

Table 3 represents the CLARK analysis results. You can find additional files in the *Results* folder

Object_ID	Length	Gamma	1st assign	score1	2nd assign	score2	Conf.
000029F|arrow	2945	0.0188034	471223	3	214473	3	0.5
000004F|arrow	42937	0.0656383	9	42	1491	33	0.56
000028F|arrow	3234	0.0821406	1491	11	2183896	10	0.52381
000027F|arrow	3428	0.0234742	1502	5	1870984	4	0.55555
000013F|arrow	15435	0.0607201	9	20	1502	15	0.571429
000019F|arrow	9196	0.0252833	232259	6	984	5	0.545455
000008F|arrow	44732	0.0092145	1464	115	2208	109	0.513393
000005F|arrow	45869	0.052891	9	56	1491	38	0.595745
000006F|arrow	40084	0.0108327	1464	62	2208	45	0.579439
000010F|arrow	19966	0.0593101	9	21	1491	14	0.6
000024F|arrow	3809	0.0187385	1260	3	1903686	3	0.5
000023F|arrow	3331	0.0111749	2421	3	169679	3	0.5
000009F|arrow	34672	0.0616415	1491	64	9	37	0.633663
000022F|arrow	3832	0.0188877	603050	3	1260	3	0.5
000020F|arrow	6708	0.0820873	1491	17	1501	12	0.586207
000014F|arrow	12150	0.0445177	9	10	28197	7	0.588235
000001F|arrow	121947	0.0704356	9	223	1491	171	0.56599
000012F|arrow	34621	0.0663276	76853	67	326544	55	0.54918
000018F|arrow	9568	0.0101592	1464	23	2208	8	0.741935
000026F|arrow	2661	0.0488451	1496	6	1986224	6	0.5
000021F|arrow	4119	0.0292754	897	5	28446	3	0.625
000031F|arrow	4313	0.0118798	2058137	6	32054	5	0.545455
000015F|arrow	12510	0.065012	1750719	25	9	24	0.510204
000025F|arrow	5012	0.0550881	84377	5	2173169	4	0.555556
000000F|arrow	133877	0.0683416	1491	1224	9	460	0.726841
000003F|arrow	66898	0.0125602	1464	211	2208	171	0.552356
000011F|arrow	23746	0.0258788	49118	12	1505	9	0.571429
000016F|arrow	16102	0.0688969	9	25	1491	24	0.510204
000030F|arrow	2328	0.0324957	363852	3	9	3	0.5
000017F|arrow	9380	0.00726496	1464	22	2208	5	0.814815
000007F|arrow	49772	0.0514351	9	54	1491	41	0.568421
000002F|arrow	61748	0.0635044	9	74	1491	66	0.528571

Table 3. Unfiltered results from the CLARK analysis.

5 2nd genome assembly

We could not identify any bacterial sequences from the first test assembly. As a next step, we recommended the continuation of the project. After receiving the second dataset steps were repeated like in section 2, 3 and 4.

The genome was assembled into 455 contigs using HGAP4 pipeline with strict parameters.

6 Filter contaminations

The contamination was checked again using the same approach as before. The only difference was that a few bigger contigs were split into smaller ones because the tool could not handle them.

You can find the tables that contains the detailed results in the *Results* folder.

7 Quality assessment

The quality assessment was done using QUAST v4.4. Table 4 contains the main metrics of the assembly and Figure 2 shows the cumulative lengths of the contigs. You can find additional files in the *Results* folder.

Numer of contigs (>= 0 bp)	455
Total length (>= 0 bp)	230,236,420
Total length (>= 25000 bp)	229,447,404
Total length (>= 50000 bp)	22,026,441
Total number of contigs	455
Largest contig	12,224,306
Total length	230,236,420
GC (%)	30.49
N50	30,655,30
N75	1,046,560
L50	20
L75	49
Numer of N's per 100 kbp	0.00

Table 4. Summary of the assembly metrics.

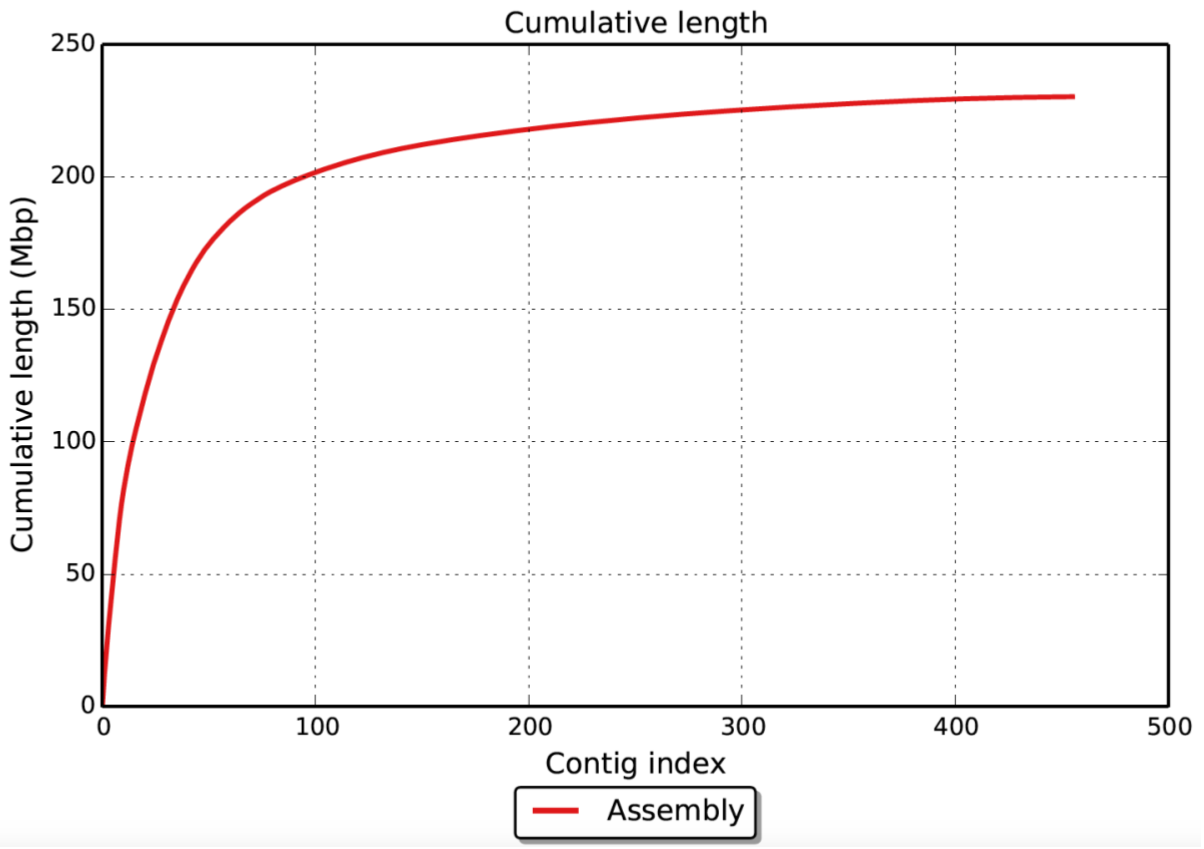

Figure 2. Cumulative length plot of contigs.

Additional quality assessment was done using BUSCO v2 [4] against the metazoa_odb9 (eukaryota) database in order to get information about the completeness of the draft assembly based on the identified core genes. Figure 3 represents the BUSCO results.

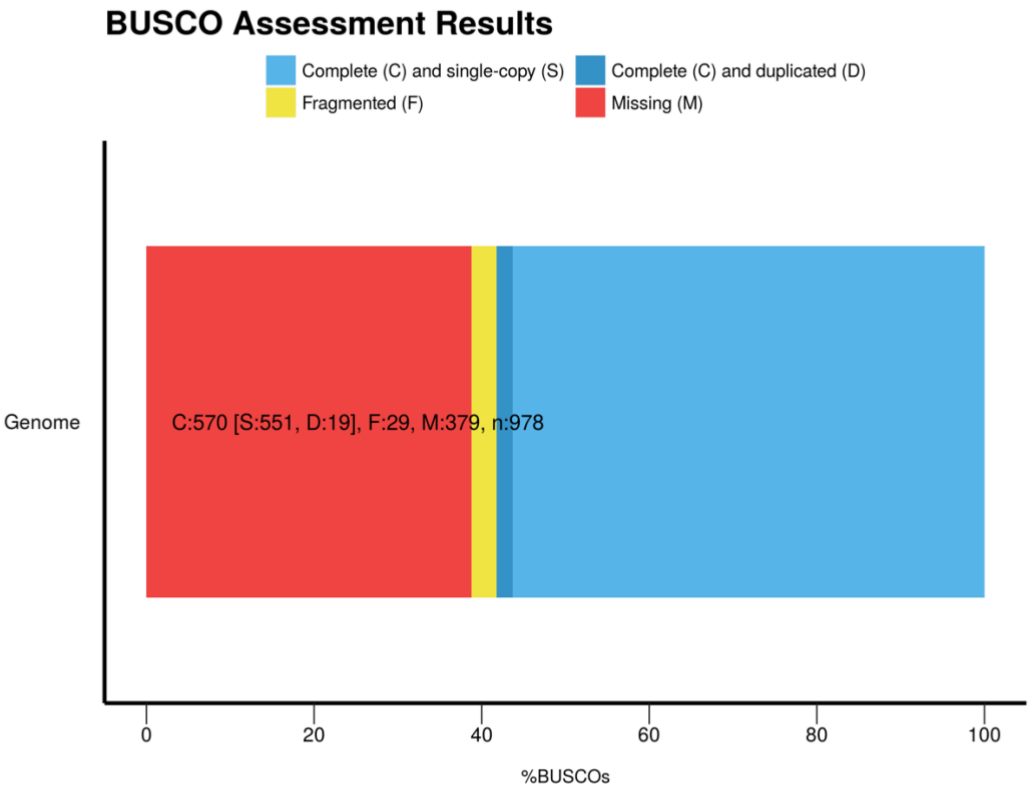

Figure 3. BUSCO results.

8 Summary of the result files

The result files can be accessed by opening the *Results* directory. You can download the result from the link below:

https://drive.google.com/drive/folders/1zt1cwYFxUcl6_zejRfevMpArCjoWz3UN?usp=sharing

Please make sure that you have downloaded all results.

The following table lists the different folders and files found in the result package.

Main Folder	*File*	*Description*
genome_fasta	genome.fasta	FASTA file contains the assembled contigs.
quast_results_1/2	report.txt	Assessment summary in plain text format.
	report.tsv	Tab-separated version of the summary.
	report.tex	LaTeX version of the summary.
	icarus.html	Icarus main menu with links to interactive viewers.
	report.pdf	All other plots combined with all tables.
	report.html	HTML version of the report with interactive plots inside.
	Subfolders	*Description*
	basic_stats	Separate plots in PDF format.
	icarus_wievers	Links of interactive viewers.
clark_1/2	_filtered.xlsx	Filtered CLARK results.
	_unfiltered.xlsx	Unfiltered CLARK results.
	_raw.xlsx	Raw CLARK results.
busco	short_summary.txt	Brief summary about BUSCO run.
	full_table.tsv	Detailed BUSCO run.
	missing_busco_list.tsv	List of missing BUSOC genes.
	busco_figure.png	BUSCO figure.

References

[1] Alla Mikheenko, Gleb Valin, Andrey Prjibelski, Vladislav Saveliev, Alexey Gurevich, Icarus: visualizer for de novo assembly evaluation, Bioinformatics (2016) 32 (21): 3321-3323. doi: 10.1093/bioinformatics/btw379 First published online: July 4, 2016

[2] Chin, et al. (2013). Nonhybrid, finished microbial genome assemblies from long-read SMRT sequencing data. Nature Methods. 10(6), 563.

[3] Ounit R, Wanamaker S, Close TJ, Lonardi S, CLARK: fast and accurate classification of metagenomic and genomic sequences using discriminative k-mers. BMC Genomics 2015, 16:236. DOI: 10.1186/s12864-015-1419-2

[4] BUSCO: assessing genome assembly and annotation completeness with single-copy orthologs. Felipe A. Simão, Robert M. Waterhouse, Panagiotis Ioannidis, Evgenia V. Kriventseva, and Evgeny M. Zdobnov Bioinformatics, published online June 9, 2015 doi: 10.1093/bioinformatics/btv351
