# Supplementary material for: Comparative analysis reveals within-population genome size variation in a rotifer is driven by large genomic elements with highly abundant satellite DNA repeat elements
Source: BMC Biol. 2021 Sep 16;19:206. doi: 10.1186/s12915-021-01134-w (PMC8447722; doi:10.1186/s12915-021-01134-w)
Supplement: Supplementary file 7 — Additional file 7. Repeat profile, Gene density, and CNVs of the 50 largest contigs. [file 12915_2021_1134_MOESM7_ESM.html]

Combined\_CNV\_Repeats\_Analysis


# Combined\_CNV\_Repeats\_Analysis

This file contains a combined analysis of repeat content, gene density, and CNVs of the largest 50 contigs of the B. asplanchnoidis genome.

Plotting the data:
